# Fibroblast growth factor 10 attenuates advanced liver fibrosis through hepatocyte fibroblast growth factor receptor 2 signalling

**DOI:** 10.1002/ctm2.70675

**Published:** 2026-05-12

**Authors:** Xuanxin Yang, Qingqing Dong, Jiaying Ma, Yawei Yan, Shuangyan Peng, Qiqi Wu, Huan Wang, Tingting Zhang, Panyu Zhang, Zelong Jiang, Chao Lu, Le Li, Xinyi You, Yuandong Xu, Joshua Banda, Zhixiang Mu, Minghua Zheng, Xiaokun Li, Qi Hui, Bingjie Yu, Xiaojie Wang

**Affiliations:** ^1^ School of Pharmaceutical Sciences Oujiang Laboratory (Zhejiang Lab for Regenerative Medicine, Vision, and Brain Health) Wenzhou Medical University Wenzhou China; ^2^ Institute for Safflower Industry Research of Shihezi University/Pharmacy College of Shihezi University/Key Laboratory of Xinjiang Phytomedicine Resource and Utilization Ministry of Education Shihezi China; ^3^ MAFLD Research Center Department of Hepatology the First Affiliated Hospital of Wenzhou Medical University Wenzhou China; ^4^ Key Laboratory of Diagnosis and Treatment for the Development of Chronic Liver Disease in Zhejiang Province Wenzhou China

**Keywords:** fibroblast growth factor 10, fibroblast growth factor receptor 2, hepatic stellate cells, hepatocyte, liver fibrosis, metabolic dysfunction–associated steatotic liver disease, steatohepatitis

## Abstract

**Background:**

Metabolic dysfunction–associated steatotic liver disease (MASLD) and advanced fibrotic stages are significant contributors to cirrhosis and liver‐related mortality, yet no therapies directly target fibrosis in the later stages of the disease. Fibroblast growth factor 10 (FGF10) facilitates epithelial repair, yet its function and epithelial receptor requirements in chronic liver fibrogenesis are unclear.

**Methods:**

We quantified hepatic FGF10 and fibroblast growth factor receptor 2 (FGFR2) expression across fibrosis stages in biopsies from patients with MASLD and mouse models. We then augmented hepatic FGF10 using adeno‐associated virus‐mediated liver expression or subcutaneous recombinant human FGF10 in carbon tetrachloride (CCl_4_) and high‐fat diet plus CCl_4_‐induced advanced fibrosis. Histology, immunohistochemistry, biochemistry, RNA sequencing and primary hepatocytes and hepatic stellate cells (HSCs) assays were used to assess the therapeutic effects and underlying mechanisms.

**Results:**

Hepatic FGF10 and FGFR2 protein expression were significantly reduced at advanced disease stages. Restoring FGF10 led to regression‐associated remodelling of established bridging fibrosis, a decrease in inflammatory cytokines and a reduction in hepatocyte apoptosis, even with continued CCl_4_ exposure, indicating histologic regression rather than slowed progression. These therapeutic effects required hepatocyte FGFR2, as hepatocyte‐specific FGFR2 deletion abolished protection and the associated transcriptional reprogramming of matrix and cytokine networks. In primary hepatocytes, FGF10 activated FGFR2–FGFR substrate 2α (‌FRS2α) signalling, increased inhibitory phosphorylation of glycogen synthase kinase 3β at Ser9 and suppressed nuclear factor kappa B, thereby lowering transforming growth factor β1 and other cytokines and indirectly limiting HSC activation. The efficacy extended to a high‐fat diet plus CCl_4_ model of steatohepatitis.

**Conclusions:**

These findings elucidate a hepatocyte‐centric FGF10–FGFR2 axis functioning as an epithelial regulator of inflammation and fibrogenesis. Hepatocyte‐targeted reinforcement of FGFR2 signalling, alone or combined with metabolic therapies, represents a translational strategy to reprogram the fibrotic niche and facilitate fibrosis regression‐associated architectural remodelling in advanced liver fibrosis.

**Key points:**

- Hepatic FGF10 and FGFR2 decline consistently with fibrosis stage in patient liver biopsies and complementary mouse models.
- Restoring FGF10 through AAV‐Fgf10 or recombinant FGF10 reduces hepatocyte apoptosis, inflammation and extracellular matrix deposition.
- Hepatocyte FGFR2 is required for FGF10‐mediated antifibrotic effects and drives transcriptional reprogramming that limits fibrogenesis.

## Graphical abstract

1. FGF10‐FGFR2 signaling is progressively impaired during liver fibrosis.
2. FGF10 restoration protects hepatocytes from inflammatory and apoptotic injury.
3. Hepatocyte FGFR2 mediates FGF10‐driven suppression of fibrogenic remodeling through the GSK3β/NF‐κB axis.

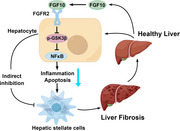

## 1 INTRODUCTION

Metabolic dysfunction–associated steatotic liver disease (MASLD) has emerged as the most prevalent chronic liver disorder, impacting approximately 30% of adults globally.^1^, ^2^, ^3^ While simple steatosis is frequently asymptomatic, a subset of individuals advances to metabolic dysfunction–associated steatohepatitis (MASH), characterized by inflammation, ballooning injury and, crucially, progressive fibrosis.^4^, ^5^, ^6^ Within the spectrum of MASLD, the degree of hepatic fibrosis serves as the most significant histological indicator of both liver‐related and overall mortality. However, there remains a lack of approved therapies specifically targeting fibrosis, particularly in its advanced stages.^7^, ^8^, ^9^ The increasing prevalence of MASLD, coupled with this therapeutic deficiency, highlights the critical need to identify molecular pathways that not only drive fibrotic progression but can also be leveraged to arrest or reverse advanced fibrosis.

Liver fibrosis is initiated by hepatocyte injury and perpetuated through the paracrine activation of hepatic stellate cells (HSCs). Injured hepatocytes release inflammatory mediators and undergo apoptosis, processes governed by intracellular signalling pathways such as nuclear factor kappa B (NF‐κB) and glycogen synthase kinase 3β (GSK3β).^10^, ^11^, ^12^ This pathological state leads to an increased secretion of transforming growth factor‐β1 (TGF‐β1) and other cytokines,^10^, ^13^, ^14^ which facilitate the transdifferentiation of HSCs into myofibroblast‐like cells responsible for the production of extracellular matrix (ECM).^15^, ^16^, ^17^ From a translational standpoint, interventions targeting hepatocytes to restore regulatory control represent promising opportunities for antifibrotic therapy.

Fibroblast growth factors (FGFs) play a crucial role in orchestrating development, tissue homeostasis and repair across various organs. In the liver, endocrine FGFs, such as FGF19 and FGF21, are involved in the regulation of bile acid and metabolic homeostasis and are currently under investigation as potential therapeutic agents for MASLD.^18^, ^19^, ^20^ In contrast, the role of paracrine FGFs in chronic liver injury remains less well‐characterized. FGF10, a representative paracrine FGF, primarily signals through epithelial fibroblast growth factor receptor 2 (FGFR2),^21^, ^22^, ^23^ and facilitates tissue regeneration.^24^, ^25^ However, its role in chronic liver fibrogenesis and the necessity for hepatocyte FGFR2 are not yet fully elucidated. Importantly, FGF10 has been shown to reduce apoptosis and mitigate inflammatory signalling in extrahepatic tissues,^26^, ^27^ suggesting that an epithelial FGF10–FGFR2 axis may function as an intrinsic mechanism to modulate injury‐induced inflammation in the liver.

Our study identified a reduction in hepatic FGF10 and FGFR2 expression in MASLD, particularly in cases of advanced fibrosis, as observed in both patient liver biopsies and murine models. This finding aligns with existing literature indicating that FGF10 mitigates apoptosis and attenuates inflammatory signalling in various tissues, suggesting that the FGF10–FGFR2 axis may function as an intrinsic epithelial mechanism to curb injury‐induced inflammation. We hypothesized that chronic hepatic injury leads to the downregulation of the FGF10–FGFR2 axis within hepatocytes, thereby removing a cytoprotective barrier against inflammatory and apoptotic signalling and enhancing paracrine activation of HSCs. Furthermore, we proposed that enhancing FGF10 levels could suppress inflammation and apoptosis in a manner dependent on hepatocyte FGFR2, resulting not only in the inhibition of fibrosis but also in architectural remodelling associated with the regression of established fibrosis, even at the bridging fibrosis stage, which is of significant clinical relevance to MASLD/MASH. Consequently, we explored the hepatocyte FGF10–FGFR2 axis as a hepatocyte‐focused therapeutic target to restore epithelial regulation of the fibrotic niche in advanced liver fibrosis and to develop a translational framework for epithelial FGFR2‐targeted antifibrotic interventions.

## 2 MATERIALS AND METHODS

### 2.1 Human liver biopsy studies

Liver tissue samples were collected from patients diagnosed with MASLD at the First Affiliated Hospital of Wenzhou Medical University and stratified by fibrosis stage (FS0–FS3) using blinded histopathological scoring (FS0, *n* = 10; FS1, *n* = 9; FS2, *n* = 8; FS3, *n* = 4). Formalin‐fixed, paraffin‐embedded (FFPE) sections were employed for quantitative immunohistochemistry to assess FGF10 abundance and localization. Fresh biopsy material (FS0–FS2) was used for RNA extraction and quantitative real‐time polymerase chain reaction (qRT‐PCR). The study protocol received approval from the institutional ethics committee (approval no. 2016/246); and written informed consent was obtained from all participants. Importantly, no samples were obtained from executed prisoners or institutionalized individuals. A summary of clinical characteristics is provided in Table S1.

### 2.2 Mice and housing

Male C57BL/6J mice (aged 5–8 weeks) were maintained under specific pathogen‐free conditions (23 ± 2°C; 60 ± 5% humidity; 12‐h light/dark cycle) with ad libitum access to standard chow and water. All experimental procedures were approved by the Animal Ethics Committee of Wenzhou Medical University (approval no. wydw2022‐0691) and adhered to the guidelines outlined in the Guide for the Care and Use of Laboratory Animals. Mice of the same genotype were randomized to treatment groups; investigators were blinded to group assignment during histologic and image analyses unless stated otherwise.

### 2.3 Diet‐induced model

Eight‐week‐old male mice were fed a 60% kcal high‐fat diet (HFD) for up to 20 weeks, with liver samples collected at Weeks 5, 16 and 20.

### 2.4 Carbon tetrachloride (CCl_4_)‐induced fibrosis

Eight‐week‐old male mice received intraperitoneal CCl_4_ (.5 mL/kg; 25% in olive oil) three times weekly for 6 weeks, with controls receiving a vehicle. Samples were collected at Weeks 0, 1, 2, 4 and 6 for analysis.

### 2.5 HFD plus CCl_4_ model

Eight‐week‐old male mice were fed an HFD for 20 weeks, with CCl_4_ administered during the final 8 weeks at the above dose/schedule to induce bridging fibrosis.

### 2.6 Liver‐directed FGF10 overexpression

Hepatotropic overexpression was achieved using an AAV2/8 vector encoding mouse *Fgf10* under the control of a CAG promoter (AAV‐*Fgf10*), alongside an AAV‐GFP control vector. Five‐week‐old male mice were administered 1.2 × 10^11^ vector genomes via the lateral tail vein in a 100 µL volume. Mice were allowed a 3‐week recovery period prior to the induction of fibrosis. Detailed vector information, including lot numbers, is available in the Key Resources Table (KRT).

### 2.7 Hepatocyte‐specific Fgfr2 knockout

*Fgfr2* ^flox/flox^ (*Fgfr2^FF^*) mice were bred with Alb‐Cre mice to generate hepatocyte‐specific knockout mice (*Fgfr2^LKO^*). PCR‐based genotyping of toe tissue from 3‐week‐old male mice confirmed the successful generation of *Fgfr2^LKO^* mice, as depicted in Figure S13. The sequences of the primers are provided in Table S2. The efficiency of knockout at the protein level in the liver was validated in 8‐week‐old male mice through western blot analysis using anti‐FGFR2 antibodies, as detailed in Table S3. Age‐matched *Fgfr2^LKO^* and *Fgfr2^FF^* male mice were subjected to CCl_4_‐induced injury as previously described.

### 2.8 Administration of recombinant human FGF10 in vivo

During the CCl_4_‐induced injury, mice received a daily subcutaneous injection of rhFGF10 (1, 2, or 5 mg/kg in  .9% saline; 5 µL/g) on the dorsal surface, commencing after 2 weeks of CCl_4_ exposure and continuing throughout the remaining injury period, unless specified otherwise. Control mice were administered the vehicle. The endotoxin content of rhFGF10 was less than 5 EU/mg, and its bioactivity was measured at 2.8 × 10^5^ AU/mg.

### 2.9 In vivo optical imaging

To assess liver‐specific transduction, mice were administered either AAV‐GFP or saline and were subsequently imaged at 0–4 weeks using an IVIS Lumina III system under isoflurane anaesthesia. Fluorescence was captured with 480 nm excitation and 520 nm emission using identical settings. At the conclusion of the 4 weeks, major organs were harvested for ex vivo imaging under matched exposure conditions. Radiant efficiency was quantified using Living Image software and visualized with the “YellowHot” colormap, applying individualized scale limits.

### 2.10 Serum biochemistry and organ indices

Mice were anesthetized with an intraperitoneal injection of pentobarbital at a dosage of 100 mg/kg. Blood samples were collected from the retro‐orbital sinus, allowed to clot and then centrifuged at 3000 × *g* for 30 min at 4°C. Serum levels of alanine aminotransferase (ALT) and aspartate aminotransferase (AST) were determined using commercial assay kits. Body weight was measured immediately prior to terminal procedures. Following cardiac perfusion with saline, livers were excised, photographed against a standardized background, weighed and the liver‐to‐body‐weight ratio was calculated.

### 2.11 Histology and morphometry

Liver tissue was fixed in 4% paraformaldehyde, processed into FFPE blocks and sectioned at a thickness of 5 µm for haematoxylin and eosin (H&E) and Sirius Red (SR) staining, adhering to standard protocols. For lipid staining, the liver tissue was embedded in optimal cutting temperature compound, rapidly frozen and cryosectioned at a thickness of 10 µm for Oil Red O staining. Images were acquired using a Nikon ECLIPSE NI microscope and analysed with Image‐Pro Plus software. Fibrosis quantification was performed by assessing the SR‐positive area fraction and/or by stage scoring, as detailed in figure legends. Observers were blinded to the experimental group.

### 2.12 Immunohistochemistry

FFPE liver sections (5 µm) were deparaffinized, rehydrated and subjected to heat‐induced antigen retrieval in  .01 M citrate buffer (pH 6.0) for 20 min. Endogenous peroxidase activity was inhibited using 3% hydrogen peroxide (H_2_O_2_) for 10 min. Following a blocking step with 5% goat serum in phosphate‐buffered saline (PBS) for 30 min, sections were incubated overnight at 4°C with primary antibodies. Detailed information regarding all antibodies, including their sources and dilutions, is provided in KRT and Table S3. Detection was conducted utilizing the commercially available SABC‐POD (rabbit IgG) kit, following the manufacturer's protocol, which involved a 30‐min incubation at 37°C with a biotinylated anti‐rabbit secondary antibody, followed by the SABC‐POD complex under identical conditions. Visualization was achieved using the kit‐supplied DAB substrate. Slides were counterstained with haematoxylin, subjected to dehydration and mounted with coverslips. Subsequently, imaging was carried out using a Nikon ECLIPSE Ni microscope, and analysis was performed with Image‐Pro Plus software. Quantification was executed through Colour Deconvolution (H‐DAB), employing a consistent threshold across all images within the same batch.

### 2.13 Immunofluorescence

Following deparaffinization and rehydration, sections underwent antigen retrieval as previously described. Permeabilization was achieved using  .1% Triton X‐100 for 10 min, followed by blocking with 5% BSA in PBS for 30 min at 37°C. Primary antibodies were incubated overnight at 4°C, succeeded by a 1‐h incubation with fluorophore‐conjugated secondary antibodies at 37°C in the dark. Detailed information regarding antibody sources and dilutions is provided in KRT and Table S3. Nuclei were counterstained with DAPI, and slides were mounted using an antifade medium. Imaging was conducted using a Zeiss LSM 980 laser‐scanning confocal microscope under consistent acquisition settings. The immunofluorescence (IF) staining protocol, including primary antibody dilutions and imaging parameters, was optimized to minimize background noise and enhance signal specificity. Quantification of mean fluorescence intensity was executed using automated measurements in Zeiss ZEN software, maintaining uniform parameters across all groups. For cultured cells, fixation was performed with 4% paraformaldehyde for 15 min, at room temperature prior to permeabilization and blocking, with subsequent staining steps mirroring those used for tissue sections.

### 2.14 Terminal deoxynucleotidyl transferase dUTP nick end labelling staining

Apoptotic nuclei were identified utilizing the one‐step terminal deoxynucleotidyl transferase dUTP nick end labelling (TUNEL) apoptosis assay kit. FFPE liver sections, with a thickness of 5 µm, underwent deparaffinization and rehydration processes. Subsequently, the sections were treated with proteinase K at a concentration of 20 µg/mL for 15 min at room temperature, followed by rinsing in PBS. The TUNEL reaction mixture was then applied for 1 h at 37°C in the dark, succeeded by additional PBS washes. The slides were counterstained with DAPI and mounted using an antifade medium. Imaging was conducted using a Zeiss LSM 980 confocal microscope, ensuring consistent acquisition settings across all experimental groups. The mean fluorescence intensity of TUNEL‐positive signals was quantified through automated measurement using Zeiss ZEN software with standardized parameters, and the data are expressed as relative values.

### 2.15 Bulk RNA sequencing and enrichment analyses

Total RNA was extracted utilizing TRIzol reagent, quantified via a NanoDrop 2000 spectrophotometer and evaluated with an Agilent 5300 Bioanalyzer. RNA samples selected for library preparation met the following quality criteria: an OD260/280 ratio between 1.8 and 2.2, an OD260/230 ratio ≥2.0, an RNA quality number ≥6.5 and a 28S:18S ratio ≥1.0, as per the standards of Majorbio Bio‐pharm Biotechnology Co., Ltd., Shanghai, China. Sequencing was executed on an Illumina NovaSeq X Plus platform (PE150). Sequence reads were aligned to the GRCm39 mouse reference genome using HISAT2 (version 2.2.1) and assembled with StringTie (version 2.2.0). Differential gene expression was analysed using DESeq2 (version 1.42.0) with Wald tests; genes exhibiting an absolute log2 fold change (|log2FC|) of  .58 or greater and a false discovery rate (FDR) of less than  .05 were classified as differentially expressed, unless specified otherwise. Gene Ontology (GO) enrichment was conducted using GOATOOLS (version 1.4.4), and pathway enrichment analysis was performed using SciPy‐based scripts. All RNA‐seq procedures and bioinformatics analyses for the tissue samples were carried out by Shanghai Majorbio Bio‐pharm Technology Co., Ltd.

### 2.16 Analysis of publicly available Gene Expression Omnibus datasets

Transcriptomic datasets available in the public domain were sourced from the NCBI Gene Expression Omnibus (GEO). Specifically, bulk RNA‐sequencing data from human liver tissues were acquired from dataset GSE246221, which includes samples from four healthy donors and 16 patients diagnosed with MASH. These patients were characterized by defined nonalcoholic fatty liver disease activity scores (NAS = 5–6) and fibrosis stages (FS = 1–3).^28^ For the GSE246221 dataset, we utilized the processed expression matrices provided by the original authors, adhering to the quality control protocols outlined in their publication. Additionally, single‐nucleus RNA‐sequencing (snRNA‐seq) data were retrieved from dataset GSE212837, encompassing three control and nine MASH liver samples exhibiting advanced fibrosis.^29^ Due to the exclusion of lowly expressed genes, including *FGF10*, in the processed h5ad objects, analyses were conducted using the original CellRanger filtered feature‐barcode matrices. Quality control was performed using Seurat (versions 4 and 5), retaining nuclei with 500–8000 detected genes (nFeature_RNA), 1000–30 000 total unique molecular identifier (UMI) counts (nCount_RNA) and a mitochondrial transcript percentage (percent.mt) of ≤5%. FGF10‐positive nuclei were characterized as those exhibiting raw counts > 0. The overall hepatic *FGF10* expression across different fibrosis stages was quantified using a pseudo‐bulk approach method, which involved aggregating raw counts within each stage.

### 2.17 Isolation and culture of primary mouse hepatocytes and HSCs

Primary mouse hepatocytes and HSCs were isolated from 8‐week‐old male mice via in situ collagenase perfusion followed by gradient centrifugation as previously described.^30^ Specifically, following the in situ collagenase perfusion, liver tissue was carefully dissociated and filtered. Hepatocytes were isolated through low‐speed centrifugation, while HSCs were obtained from the supernatant using density‐gradient centrifugation. Hepatocytes were cultured in high‐glucose Dulbecco's modified Eagle medium (DMEM) with 10% foetal bovine serum (FBS) on collagen‐coated plates and identified via albumin immunostaining. HSCs were cultured under similar conditions and identified using desmin, α‐smooth muscle actin (α‐SMA) and vitamin A autofluorescence. To reduce variability associated with cell passage, primary hepatocytes were limited to the first passage, whereas HSCs were utilized between passages one and four.

### 2.18 In vitro analysis of CCl_4_‐induced cellular injury and rhFGF10 treatment

To prepare a CCl_4_‐saturated medium, 1 mL of analytical grade CCl_4_, prefiltered through a  .22 µm nylon membrane, was transferred into an amber serum vial. Serum‐starved high‐glucose DMEM containing  .5% FBS was added to achieve a final volume of 10 mL. The vial was then sealed using a butyl rubber stopper and parafilm and incubated at 37°C for 48 h. Subsequently, the upper aqueous supernatant was collected as the CCl_4_‐saturated medium. Primary hepatocytes were exposed to this medium for either 6 or 24 h, as specified, followed by treatment with rhFGF10 at concentrations of 10 or 40 µg/mL. Vehicle controls were included to match the solvent content.

### 2.19 Annexin V‐FITC/propidium iodide staining in vitro

Hepatocytes were cultured on collagen‐coated glass‐bottom dishes and subjected to the specified treatments. Without undergoing fixation, the cells were incubated in Annexin‐V binding buffer containing Annexin V‐FITC and propidium iodide (PI) (kit; KRT) for 10 min in the dark. Subsequently, the cells were counterstained with DAPI and immediately imaged using a Zeiss LSM 980 microscope. The signals from Annexin‐V and PI were quantified using ZEN software with fixed analysis parameters.

### 2.20 RNA interference

Primary hepatocytes were transfected with either si*Fgfr2* or control siRNA employing Lipofectamine 3000 for a duration of 48 h, following the manufacturer's instructions. Subsequently, the cells were exposed to the CCl_4_‐saturated medium with or without rhFGF10 for 24 h. Knockdown efficiency was verified by immunoblotting 72 h post‐transfection (refer to Figure S15). The siRNA sequences are provided in Table S4.

### 2.21 Immunoblotting

Tissue or cell lysates were prepared using a lysis buffer supplemented with protease and phosphatase inhibitors. Equal amounts of protein, typically 30 µg, were resolved by sodium dodecyl sulfate–polyacrylamide gel electrophoresis (SDS‐PAGE), transferred onto polyvinylidene fluoride (PVDF) membranes and probed with primary and horseradish peroxidase (HRP)‐conjugated secondary antibodies (refer to KRT and Table S3). The bands were visualized using chemiluminescence and their intensities were quantified using Image Lab software, with normalization to GAPDH.

### 2.22 Quantitative real‐time PCR

Total RNA was isolated from liver tissues utilizing a TRIzol‐based extraction kit. The RNA was subsequently reverse‐transcribed using the HifairII cDNA Synthesis SuperMix. qRT‐PCR was then conducted employing the SYBR Green master mix on a QuantStudio 5 system. Gene expression levels were normalized to human *GAPDH* and mouse *Gapdh* using the 2^−ΔΔCt^ method. Primer sequences are detailed in Table S5.

### 2.23 Statistical analysis

Data are expressed as mean ± SEM. Comparisons between two groups were performed using two‐tailed unpaired Student's *t*‐tests. For analyses involving multiple groups, one‐way or two‐way analysis of variance was employed, followed by appropriate post hoc tests as indicated in the figure legends. A *p*‐value of less than  .05 was considered statistically significant. Details regarding the unit of analysis, number of biological replicates (*n*), randomization/blinding procedures and specific statistical tests used are provided in the figure legends. All statistical tests were two‐sided. The normality and homoscedasticity of the data were evaluated using the Shapiro–Wilk and Levene tests, respectively, to determine the suitability of parametric or nonparametric tests. Where applicable, multiple comparisons were adjusted using the Benjamini–Hochberg FDR method. Effect sizes and 95% confidence intervals are reported when pertinent.

## 3 RESULTS

### 3.1 Hepatic FGF10 and FGFR2 progressively decline with fibrosis severity in patients

**FIGURE 1:**
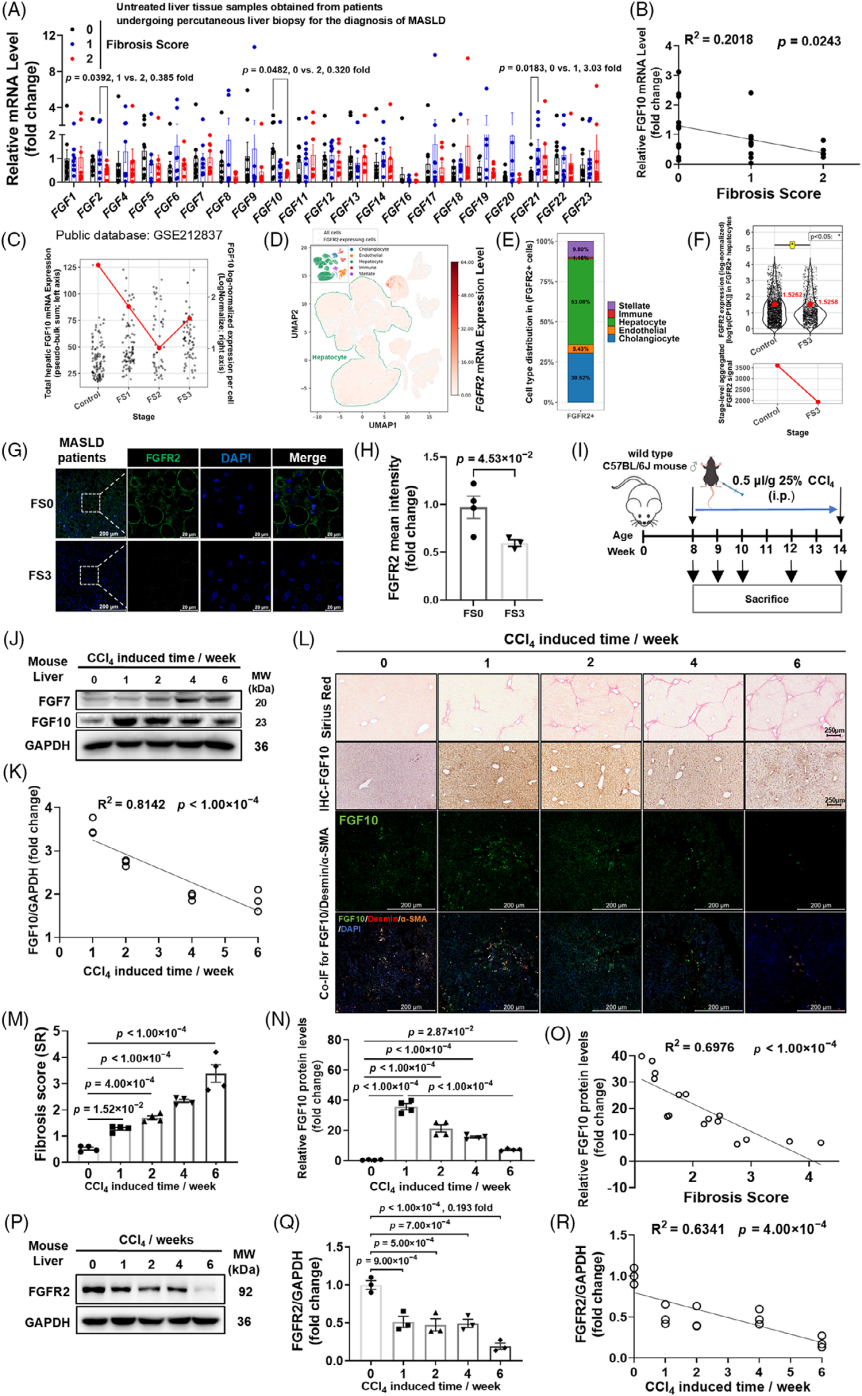
Stage‐dependent regulation of hepatic fibroblast growth factor 10 (FGF10) and fibroblast growth factor receptor 2 (FGFR2) during fibrosis progression in both patients and CCl_4_‐injured mice. (A) An analysis of hepatic *FGF* transcripts in metabolic dysfunction–associated steatotic liver disease (MASLD) biopsies categorized by fibrosis stage (FS0, *n* = 10; FS1, *n* = 9; FS2, *n* = 8). (B) Linear regression analysis of *FGF10* mRNA levels relative to fibrosis stage on a per‐patient basis (*n* = 4–10 per group across panels B–D). (C–F) Reanalysis of a publicly available human liver single‐nucleus RNA‐sequencing (snRNA‐seq) dataset (GSE212837), which includes data from three healthy controls and nine metabolic dysfunction–associated steatohepatitis (MASH) liver samples. (C) Single‐cell distribution of detectable *FGF10* transcripts across different fibrosis stages, along with aggregated total hepatic *FGF10* mRNA abundance calculated using raw transcript counts across all hepatic cells. (D) Uniform Manifold approximation and projection (UMAP) feature plot depicting *FGFR2* expression predominantly enriched in hepatocytes, based on processed snRNA‐seq data. (E) Proportional distribution of *FGFR2*‐positive cells. (F) Comparison of *FGFR2* transcript levels in hepatocytes between FS3 and healthy controls, alongside aggregated total hepatic *FGFR2* mRNA abundance at these stages. (G–H) FGFR2 immunofluorescence staining in liver sections from the same cohort, comparing FS0 (Control) and FS3, with corresponding quantification. Representative images are provided. (I) Schematic representation of the CCl_4_ protocol. (J–K) Temporal analysis of hepatic FGF10 and FGF7 protein levels via immunoblotting over a 0–6 week period (J), and regression analysis of FGF10 levels in relation to CCl_4_ exposure duration (K). (L) Representative images of Sirius Red (SR) staining, FGF10 immunohistochemistry (IHC) and immunofluorescence (IF) analyses of liver sections throughout the CCl_4_ exposure timeline. In addition to SR and FGF10 IHC, single‐channel FGF10 immunofluorescence and triple immunofluorescence staining for FGF10, along with hepatic stellate cell (HSC) markers α‐smooth muscle actin (α‐SMA) and desmin (with DAPI nuclear counterstaining), are presented. (M–N) Quantitative analysis of SR percentage (M) and FGF10 IHC (N) (*n* = 4 per group). (O) Regression analysis of FGF10 protein levels against fibrosis scores. (P–R) Temporal analysis and quantification of hepatic FGFR2 protein levels in the same CCl_4_ series. Stats: unpaired *t*‐test (F, H); one‐way analysis of variance (ANOVA) (A, M, N, Q).

We conducted a comprehensive profiling of hepatic FGF10 across various stages of fibrosis by integrating transcript and protein analyses of fresh and FFPE human liver biopsies. In the fresh liver biopsies (*n* = 27), *FGF10* mRNA expression demonstrated an inverse correlation with fibrosis stage (Figure 1A,B), whereas transcripts for *FGF2* and *FGF21* did not exhibit a linear relationship with fibrosis scores (Figure S1), underscoring the specificity of the association between FGF10 expression and fibrosis stage. A reanalysis of the human liver snRNA‐seq dataset (GSE212837) demonstrated that *FGF10* transcripts were detectable within hepatic cells at the single‐cell level. Upon aggregating raw transcript counts across all hepatic cells, a reduction in the overall abundance of hepatic *FGF10* mRNA was observed at advanced stages (Figure 1C). Quantitative immunohistochemical analysis of FFPE samples (FS0 to FS3) demonstrated a progressive decrease in FGF10 protein staining intensity correlating with the degree of fibrosis (Figure S2). Histopathological examination confirmed progressive accumulation of collagen in bridging fibrosis at stage 3 (Figure S3).

We next examined FGFR2 expression. A reanalysis of the human liver RNA‐seq dataset (GSE246221), with a NAS of 5–6, revealed a decline in *FGFR2* transcript levels corresponding to the progression of fibrosis stages from 1 to 3, while *FGFR1* did not exhibit any significant trend (Figure S4). A detailed examination of the processed snRNA‐seq dataset (GSE212837) revealed a distinctly compartmentalized expression pattern. UMAP analysis indicated that *FGFR2* expression was predominantly concentrated in hepatocytes (Figure 1D), which accounted for 53.08% of all *FGFR2*‐positive cells (Figure 1E). The mean *FGFR2* transcript levels within hepatocytes were significantly reduced in FS3 compared to healthy controls, and the overall hepatic *FGFR2* mRNA abundance was also diminished in FS3 (Figure 1F). Additionally, FGFR2 protein expression was observed to decrease with advancing fibrosis stages (FS3 versus FS0) in human biopsies (Figure 1G,H). These findings suggest a coordinated erosion of the hepatic FGF10–FGFR2 axis during the progression of human liver disease and propose its potential utility as a stage‐associated marker.

### 3.2 Hepatic FGF10 and FGFR2 are reduced at advanced stages of murine fibrosis

We first examined FGF10 in the context of diet‐induced steatosis. Mice that were fed an HFD for periods of 5, 16 or 20 weeks demonstrated slight decreases in hepatic FGF10 levels compared to chow‐fed controls, with no discernible trend related to the duration of the diet (Figure S5). This observation aligns with the absence of distinct FS3‐like bridging patterns (Figure S6). We therefore turned to a 6‐week CCl_4_ injury model (see schema in Figure 1I). In this fibrosis model, characterized by the development of FS3‐like bridging fibrosis, hepatic FGF10 protein levels were transiently elevated at 1 week but progressively declined from 1 to 6 weeks (Figure 1J,K; Figure S7A), culminating in an overall reduction at the bridging fibrosis stage (Figure 1L). In contrast, the related ligand FGF7 was upregulated and remained elevated throughout the same period (Figure 1J; Figure S7B,C). The severity of fibrosis increased with prolonged induction, and there was a strong inverse correlation between FGF10 abundance and histologic stage (Figure 1L–O). IF‐based quantification consistently demonstrated a similar temporal decline in hepatic FGF10 signal intensity during CCl_4_‐induced fibrosis (Figure S8A,B). Spatial analysis indicated that the FGF10‐positive region exhibited an expansion during the initial phase of injury (0–1 weeks), followed by a contraction in the subsequent stages (1–6 weeks; Figure S8C). Furthermore, linear regression analysis demonstrated a negative correlation between the extent of the FGF10‐positive area and the duration of fibrosis (Figure S8D). Consequently, despite an initial transient induction, hepatic FGF10 protein experiences a sustained decline as fibrosis progresses to more advanced and severe stages.

We also investigated the receptor of FGF10 and observed that hepatic FGFR2 protein levels progressively declined over the same CCl_4_ treatment time course (Figure 1P–R). In contrast, FGFR1 exhibited only a modest decrease, which plateaued at later time points (Figure S9). These results collectively align with findings from human studies and support the role of chronic injury as a catalyst for the disruption of the FGF10–FGFR2 pathway.

### 3.3 FGF10 suppresses hepatocyte inflammatory output and apoptosis and indirectly limits stellate cell activation

**FIGURE 2:**
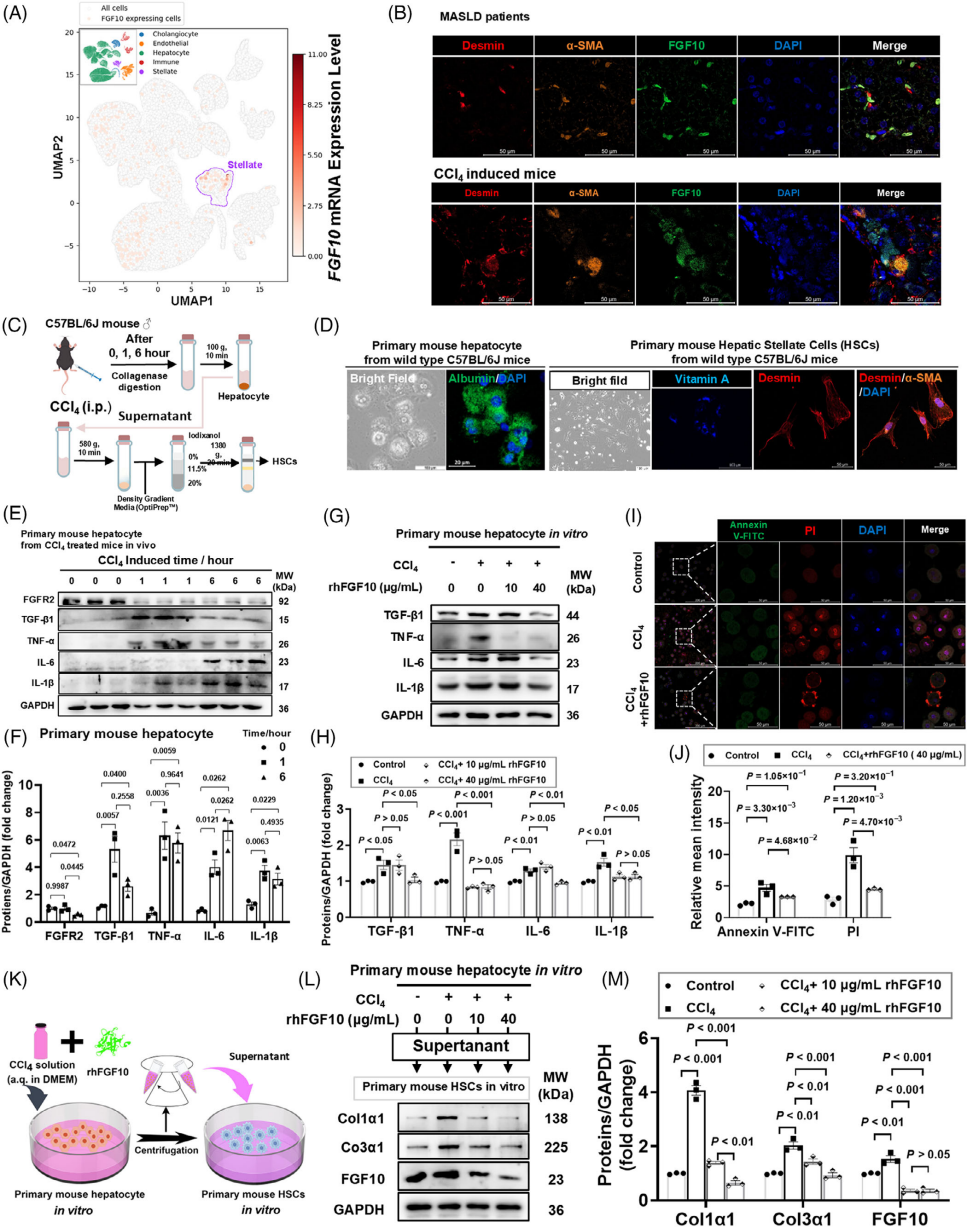
Fibroblast growth factor 10 (FGF10) attenuates the hepatocyte inflammatory response and apoptosis in hepatocytes and mitigates paracrine hepatic stellate cell (HSC) activation in vitro. (A) A reanalysis of the snRNA‐seq dataset (GSE212837) demonstrated a uniform manifold approximation and projection (UMAP) feature plot highlighting FGF10 expression within HSCs. (B) Immunofluorescence staining of human liver samples (control and FS3) and murine liver samples (sham and 6‐week CCl_4_‐injured mice) revealed colocalization of FGF10 with desmin and α‐smooth muscle actin (α‐SMA) in HSCs, including high‐magnification views and quantification of mean fluorescence intensity in individual HSCs. Scale bars, 50 µm. (C) The workflow for isolating primary cells from CCl_4_‐treated C57BL/6J mouse liver is outlined. (D) Identification of primary mouse hepatocytes (Alb+) and HSCs (characterized by vitamin A autofluorescence and desmin/α‐SMA positivity). (E–F) Immunoblot analyses of transforming growth factor β1 (TGF‐β1), TNF‐α, IL‐6, IL‐1β and fibroblast growth factor receptor 2 (FGFR2) in primary hepatocytes at 0, 1 and 6 h post‐exposure to CCl_4_ (E). Quantification is normalized to GAPDH and baseline (F, *n* = 3). (G–H) Immunoblot analyses and quantification of TNF‐α, IL‐6 and IL‐1β in hepatocytes injured by CCl_4_ and subsequently treated with varying concentrations of rhFGF10 (0, 10, 40 µg/mL) for 24 h (*n* = 3). (I–J) Annexin V/PI staining of hepatocyte apoptosis post‐rhFGF10 treatment, with representative images (I) and quantification (J) provided (*n* = 3). Scale bar, 50 µm. (K) A schematic diagram illustrates the indirect coculture system of hepatocytes and HSCs. (L–M) Immunoblot analyses and quantification of fibrogenic marker expression in HSCs cultured with hepatocyte‐conditioned media, with and without the addition of rhFGF10 (*n* = 3). Stats: one‐way analysis of variance (ANOVA) (F, H, J, M).

To elucidate the cellular origin of FGF10 during fibrogenesis, analysis of the snRNA‐seq dataset (GSE212837) indicated that *FGF10* transcripts were predominantly localized to the HSC cluster (Figure 2A). Complementing the transcript‐level findings, high‐resolution imaging revealed that FGF10 protein signals were primarily associated with desmin‐ and α‐SMA‐positive HSCs in FS3 human livers and CCl_4_‐injured mouse livers (Figure 2B). This spatial distribution was further validated by line‐scan fluorescence intensity profile analyses, which confirmed the presence of FGF10 signals in α‐SMA‐ and desmin‐positive cells during the progression of fibrosis (Figure S8E). Collectively, these findings identify HSCs as the primary cellular source of FGF10 during the process of fibrogenesis.

These findings, when considered alongside the previously documented hepatocyte‐enriched expression of FGFR2, provide evidence in favour of a paracrine signalling model. We proceeded to investigate the potential modulatory effects of FGF10 on the communication between hepatocytes and HSCs using primary cells. As depicted in the isolation protocol (Figure 2C), liver tissues were subjected to collagenase perfusion, allowing for the recovery of hepatocytes through low‐speed sedimentation, while HSCs were enriched from the supernatant using a density gradient technique. Primary hepatocytes and HSCs were validated using lineage‐specific markers (Figure 2D). In hepatocytes, acute exposure to CCl_4_ in vivo (at 0, 1 and 6 h) resulted in a rapidly increased levels of in inflammatory mediators, including TGF‐β1, TNF‐α, IL‐6 and IL‐1β, accompanied by a concurrent decrease in FGFR2 protein levels, as verified by immunoblot analysis (Figure 2E,F). Given that receptor‐binding determinants are conserved and recombinant human FGF10 (rhFGF10) effectively interacts with the murine receptor, we employed rhFGF10 for in vitro stimulation. In CCl_4_‐injured hepatocytes, rhFGF10 at concentrations of 10 and 40 µg/mL reduced levels of TGF‐β1, TNF‐α, IL‐6 and IL‐1β in a concentration‐dependent manner over 24 h, with maximal suppression observed at 40 µg/mL (Figure 2G,H). Annexin V‐FITC/PI staining indicated reduced apoptosis following rhFGF10 treatment (Figure 2I,J).

To evaluate the paracrine effects on HSCs, conditioned medium derived from hepatocytes treated with CCl_4_ with or without rhFGF10 was administered to primary HSCs for a duration of 24 h (see schema in Figure 2K). Compared to the medium obtained from hepatocytes treated solely with CCl_4_, the rhFGF10‐conditioned medium resulted in a reduction of Col1a1 and Col3a1 expression and a decrease in FGF10 levels within HSCs (Figure 2L,M; Figure S10), which is indicative of diminished HSC activation. These findings elucidate the role of hepatocyte FGF10–FGFR2 signalling as an inhibitor of hepatocyte inflammatory response and apoptosis and demonstrate its ability to exert a paracrine inhibitory effect on HSC activation.

### 3.4 Hepatic FGF10 overexpression reprograms inflammatory and profibrotic transcriptional networks

**FIGURE 3:**
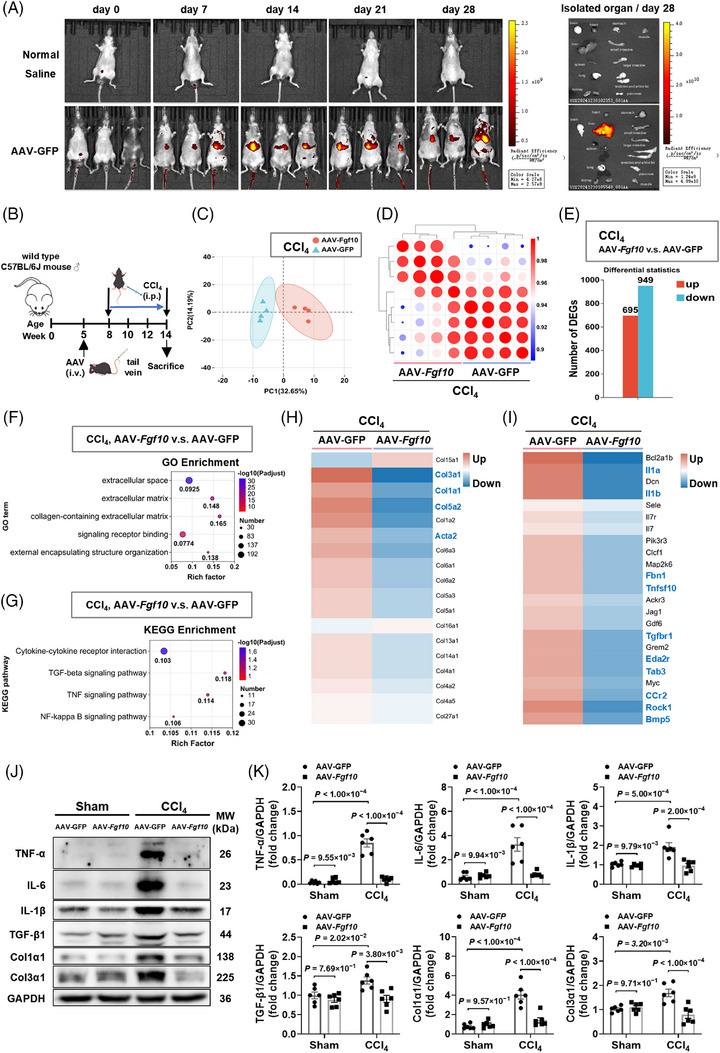
Fibroblast growth factor 10 (FGF10) reprograms inflammatory and extracellular matrix (ECM) programs in CCl_4_‐injured mouse liver. (A) In vivo and ex vivo AAV‐GFP signals were observed over a 28‐day period following intravenous administration. (B) Study design: administration occurred at Week 5, followed by CCl_4_ treatment from Weeks 8 to 14. (C–E) Principal component analysis (PCA) demonstrated separation, with replicate correlation and differential expression gene (DEG) counts comparing AAV‐*Fgf10* and AAV‐GFP groups (*n* = 4 per group). (F–G) Gene Ontology (GO) and Kyoto Encyclopaedia of Genes and Genomes (KEGG) enrichment analysis underscored the involvement of ECM and cytokine pathways, as well as transforming growth factor β (TGF‐β), TNF signalling. (H–I) Heatmaps illustrated the expression patterns of collagen, Acta2 and cytokines modules, including TGF‐β, TNF pathways. (J–K) Immunoblot analysis and quantification were performed for TNF‐α, IL‐6, IL‐1β, TGF‐β1, Col1α1 and Col3α1, comparing sham versus CCl_4_ treatment and AAV‐GFP versus AAV‐*Fgf10* groups (*n* = 6 per group). Stats: two‐way analysis of variance (ANOVA) (K), and RNA‐seq DEGs were identified with a false discovery rate (FDR) of less than  .05 and an absolute log2 fold change (|log2FC|) of at least  .58.

To evaluate the functional implications of hepatic FGF10 in vivo, we employed liver‐directed overexpression utilizing an AAV2/8 vector encoding *Fgf10* (AAV‐*Fgf10*) with AAV‐GFP serving as the control.^31^ In vivo fluorescence imaging showed a progressively increasing signal over time, with a dominant abdominal/liver‐area distribution, and ex vivo organ imaging at day 28 demonstrated predominant hepatic signal with minimal near‐background signal in non‐hepatic organs under the same acquisition settings (Figure 3A). Correspondingly, both hepatic FGF10 protein levels and *Fgf10* mRNA expression were elevated in mice treated with AAV‐*Fgf10* (Figure S11A–C). After establishing AAV expression, the mice were subjected to a 6‐week CCl_4_ injury protocol as outlined in Figure 3B. RNA‐seq of liver tissues revealed distinct group separation via principal component analysis, demonstrating high concordance within groups (Figure 3C,D). Utilizing DESeq2 with Benjamini–Hochberg correction, we identified 1644 differentially expressed genes (FDR < .05; |log2FC| ≥  .58) between the AAV‐*Fgf10* and AAV‐GFP liver samples, with 695 genes upregulated and 949 genes downregulated (Figure 3E).

GO enrichment analysis identified significant associations with collagen‐containing ECM and external encapsulating structure terms (Figure 3F). KEGG pathway analysis demonstrated alterations in cytokine‐receptor interaction and stress‐response pathways associated with TGF‐β, TNF and NF‐κB signalling (Figure 3G). Correspondingly, hierarchical clustering revealed a downregulation of fibrogenic markers, including *Col3a1*, *Col1a1*, *Col5a2* and *Acta2* (Figure 3H), and a notable reduction in representative pro‐apoptotic (e.g., *Tnfsf10*, *Rock1*, *Eda2r*) and pro‐inflammatory transcripts (e.g., *Il1a*, *Il1b*, *Ccr2*, *Tab3*) in AAV‐*Fgf10*‐treated livers (Figure 3I). Immunoblotting confirmed decreased ECM protein abundance (Col1α1, Col3α1) and reduced levels of TGF‐β1, TNF‐α, IL‐6 and IL‐1β in AAV‐*Fgf10*‐treated livers (Figure 3J,K), supporting the attenuation of profibrotic and inflammatory molecular signatures in vivo.

### 3.5 FGF10 reduces bridging fibrosis and improves liver architecture in vivo

**FIGURE 4:**
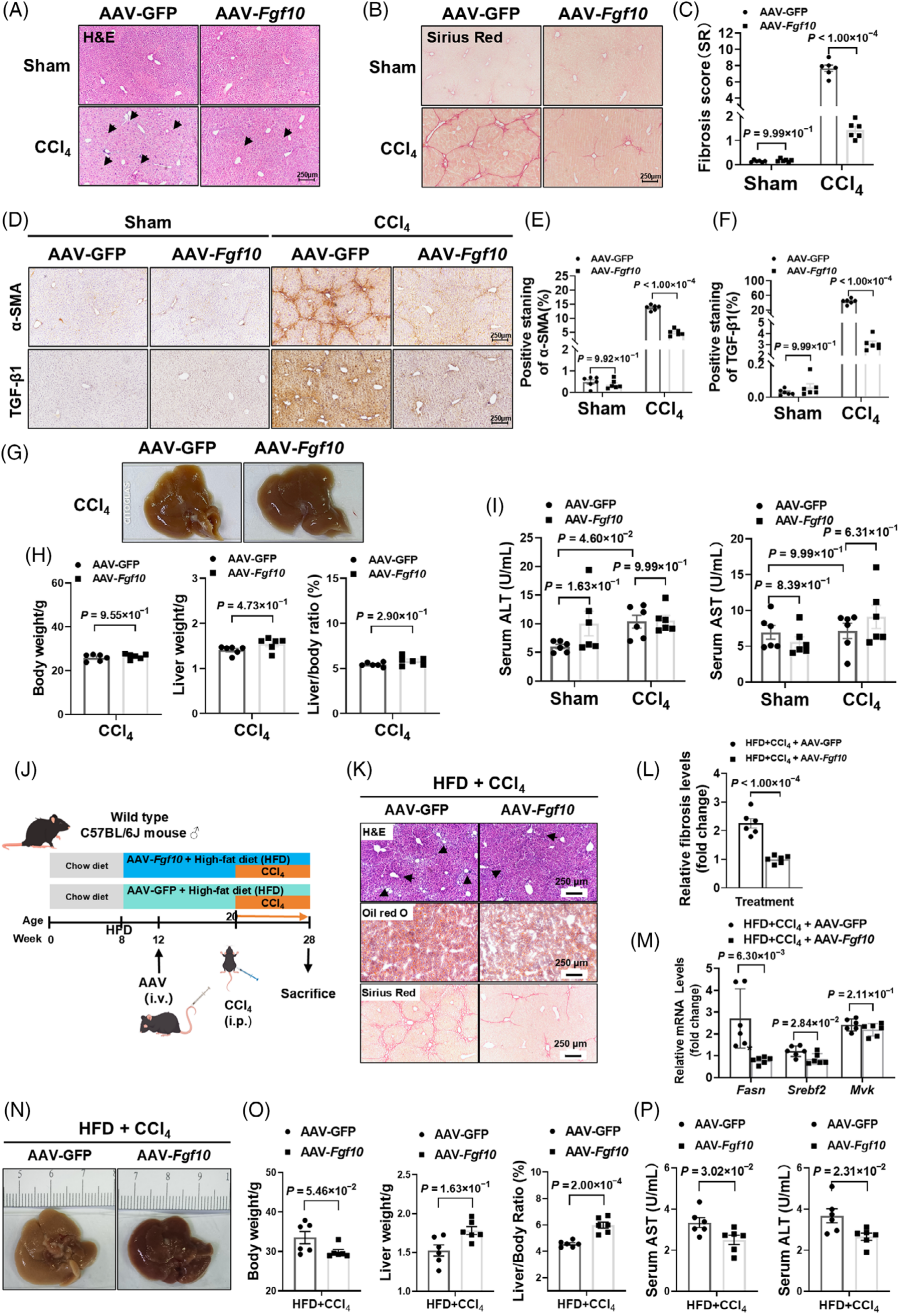
Liver‐restricted fibroblast growth factor 10 (FGF10) mitigates hepatocellular damage, inflammation and fibrosis in the context of toxicant and metabolic‐toxicant exposure. (A) Haematoxylin and eosin (H&E) staining with a scale bar of 250 µm (*n* = 6 per group). (B–C) Sirius Red (SR) staining and morphometric analysis. (D–F) Immunohistochemistry (IHC) and quantification of α‐smooth muscle actin (α‐SMA) and transforming growth factor β1 (TGF‐β1). (G–H) Gross liver imaging and assessment of body and liver indices. (I) Measurement of serum alanine aminotransferase (ALT) and aspartate aminotransferase (AST) levels. (J) Mice were administered either AAV‐*Fgf10* or AAV‐GFP at Week 5, transitioned to an HFD from Weeks 8 to 28, subjected to intraperitoneal injections of CCl_4_ from Weeks 20 to 28, and euthanized at Week 28. (K) H&E staining highlights inflammatory foci, oil red O reveals lipid accumulation and SR staining indicates collagen deposition (scale bar, 250 µm). (L) Fibrosis is quantified via SR morphometry and expressed as a fold change relative to AAV‐GFP (*n* = 6 per group). (M) Hepatic transcripts of *Fasn*, *Srebf2* and *Mvk* are reduced, as determined by quantitative real‐time polymerase chain reaction (qRT‐PCR) (*n* = 6 per group). (N–O) Gross liver morphology shows improvement, and measurements of body weight, liver weight and liver‐to‐body weight ratio are provided (*n* = 6 per group). (P) Serum levels of AST and ALT are decreased (*n* = 6 per group). Stats: unpaired *t*‐test (C, E, F, H, L, M, O, P); two‐way analysis of variance (ANOVA) (I).

We subsequently investigated the potential of hepatic FGF10 overexpression to alter histological damage and fibrogenesis. In the chronic CCl_4_ model, treatment with AAV‐*Fgf10* improved histologic architecture and decreased cellular ballooning, as evidenced by H&E staining (Figure 4A), and reduced collagen deposition on SR staining, with a concordant reduction in fibrosis score relative to AAV‐GFP controls (Figure 4B,C). Immunohistochemical analysis indicated a decrease in α‐SMA and TGF‐β1 immunoreactivity in AAV‐*Fgf10*‐treated livers, aligning with diminished HSC activation (Figure 4D–F). Systemic parameters, such as body weight, liver weight and the liver‐to‐body‐weight ratio, remained similar at the study's conclusion (Figure 4G,H), suggesting that FGF10 overexpression did not result in hepatomegaly or affect overall growth metrics. Despite persistent elevations in serum ALT and AST levels in both groups, no significant differences were observed between them (Figure 4I), indicating that biochemical normalization may lag behind histological improvement in these experimental conditions. Collectively, these data indicate that hepatocyte‐targeted FGF10 reduces collagen deposition and bridging fibrosis, thereby enhancing liver architecture in the chronic CCl_4_ model.

### 3.6 FGF10 ameliorates fibrosis and steatohepatitis in a metabolic‐toxicant injury model

In order to assess the efficacy of FGF10 within a metabolic‐toxicant framework, mice were subjected to a 20‐week HFD regimen, followed by an 8‐week administration of CCl_4_ and transduced with AAV‐*Fgf10* to facilitate hepatocyte‐specific expression of FGF10 (Figure 4J). In this model, the administration of AAV‐*Fgf10* was associated with improved parenchymal histology, reduced lipid staining and diminished SR‐positive fibrosis (Figure 4K), with significantly lower fibrosis burden by quantification (Figure 4L). AAV‐*Fgf10* also modulated lipogenic and cholesterogenic genes, including *Fasn* and *Srebf2*, with *Mvk* exhibiting a downward trend (Figure 4 M). Additionally, there was an improvement in the macroscopic appearance of the liver (Figure 4N). Although no significant differences in body weight were observed between the experimental groups, mice treated with AAV‐*Fgf10* demonstrated increased liver weight and liver‐to‐body‐weight ratio at this study endpoint (Figure 4O), indicative of the restoration of hepatic mass during resolution of injury rather than pathological enlargement. Furthermore, serum levels of AST and ALT were significantly reduced in the AAV‐*Fgf10* group (Figure 4P). At the protein level, hepatic overexpression of FGF10 suppressed profibrotic and inflammatory mediators, including Col1a1, α‐SMA, fibronectin, TNF‐α and IL‐1β, and reduced the pro‐apoptotic marker Bax (Figure S12A,B), aligning molecular, biochemical and histologic endpoints. These data collectively suggest that hepatic FGF10 functions as a suppressor of fibrosis‐associated remodelling across various injury contexts, with biochemical improvements being more pronounced in the metabolically stressed HFD + CCl_4_ model.

### 3.7 FGF10 enhances hepatic FGFR2 expression and reshapes receptor signalling in chronic fibrosis

**FIGURE 5:**
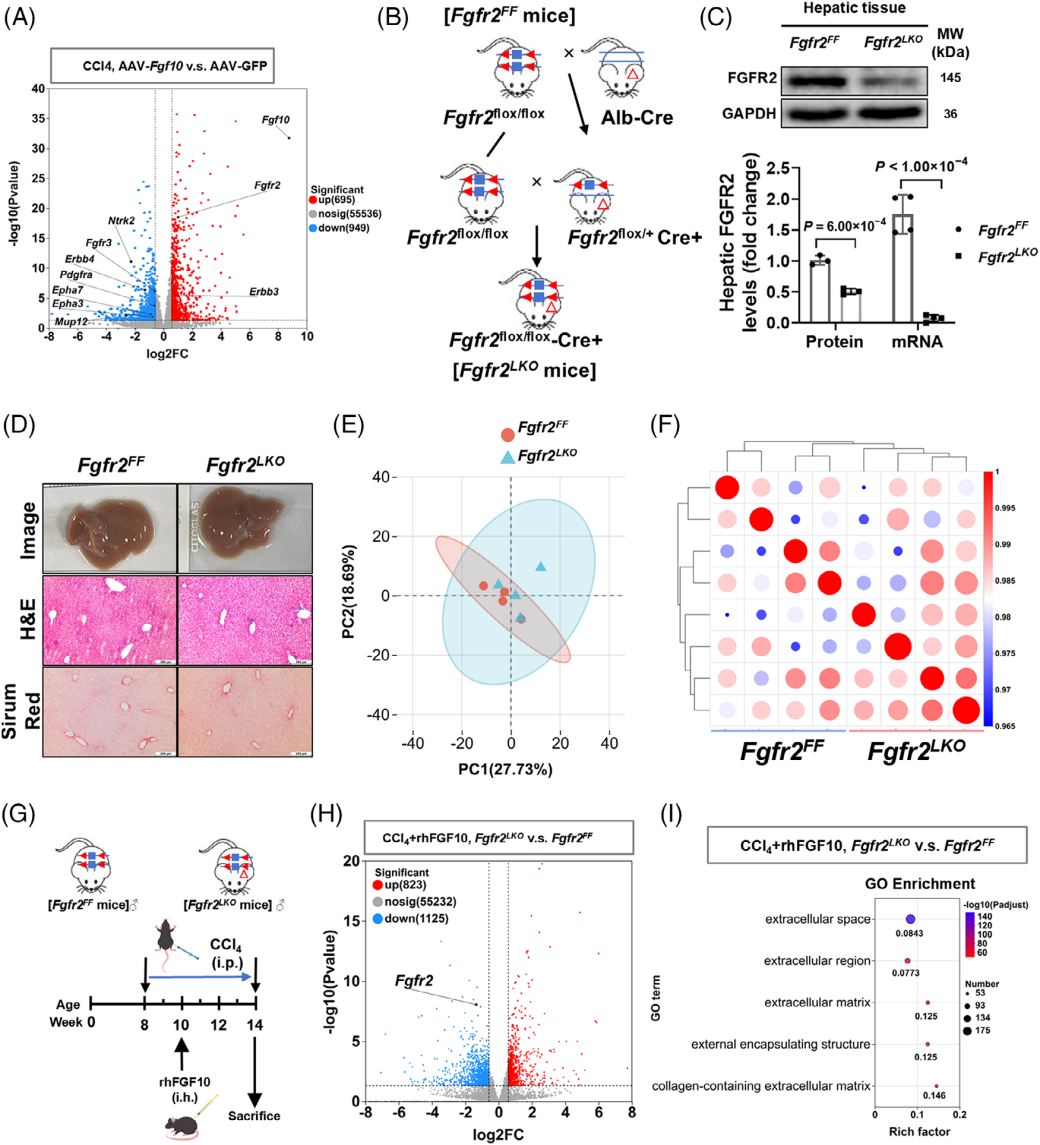
The remodelling of the fibroblast growth factor receptor 2 (FGFR2) axis under the influence of fibroblast growth factor 10 (FGF10) augmentation and the baseline characterization of hepatocyte‐specific Fgfr2 deletion were investigated. (A) A volcano plot illustrates differential expression genes (DEGs) between AAV‐*Fgf10* and AAV‐GFP groups (*n* = 4 per group). (B) The breeding strategy for *Fgfr2^LKO^* and *Fgfr2^FF^* mice is depicted. (C) The loss of hepatic FGFR2 was validated at both mRNA and protein levels in 8‐week‐old male mice. (D–F) Initial histological and transcriptomic assessment of *Fgfr2^LKO^* and *Fgfr2^FF^* mice. (D) Examination of overall liver morphology, along with haematoxylin and eosin (H&E) staining and Sirius Red (SR) staining. (E) Principal component analysis (PCA) of liver transcriptomes. (F) Analysis of sample‐to‐sample correlation. (G) The experimental design for comparing CCl_4_ and recombinant human FGF10 (rhFGF10) treatments in *Fgfr2^FF^* versus *Fgfr2^LKO^* mice is outlined. (H) An RNA‐seq volcano plot for the CCl_4_ and rhFGF10 treatment comparison between *Fgfr2^LKO^* and *Fgfr2^FF^* mice is presented. (I) Gene Ontology (GO) enrichment analysis highlights the involvement of extracellular matrix (ECM) and collagen‐containing structures. Stats: unpaired *t*‐test (C); RNA‐seq DEGs by false discovery rate (FDR) < .05.

To assess receptor‐level remodelling, we conducted a transcriptomic analysis of hepatic tissues from CCl_4_‐treated mice expressing either AAV‐*Fgf10* or AAV‐GFP (Figure 5A). As anticipated, *Fgf10* emerged as one of the most significantly upregulated transcripts, accompanied by an increase in *Fgfr2* expression. Several receptor tyrosine kinases associated with fibrogenesis, including *Pdgfra*, *Fgfr3*, *Erbb4*, *Epha3*, *Epha7*, *Ntrk2* and *Mup12*, exhibited reduced expression, whereas *Erbb3* expression was elevated. This pattern suggests a selective enhancement of the FGFR2 signalling pathway with hepatic FGF10 overexpression.

### 3.8 Establishment and validation of hepatocyte‐specific FGFR2 loss‐of‐function models

To ascertain the specificity of FGFR2‐dependent effects, we conducted genetic and molecular validation at both the animal and cellular levels. Hepatocyte‐specific FGFR2 knockout mice were generated through the crossing of *Fgfr2^flox/flox^* (*Fgfr2^FF^*) mice with Alb‐Cre transgenic mice (see schema in Figure 5B), with successful recombination confirmed via genotyping (Figure S13). Correspondingly, *FGFR2* mRNA and FGFR2 protein levels were significantly diminished in liver tissue from FGFR2 conditional knockout mice (Figure 5C). Under basal conditions, both *Fgfr2^LKO^* and *Fgfr2^FF^* mice displayed similar gross liver morphology and histology, with no signs of spontaneous fibrosis as determined by H&E and SR staining (Figure 5D).

To further elucidate the baseline hepatic phenotype associated with hepatocyte‐specific FGFR2 deletion, we conducted RNA‐seq profiling of *Fgfr2^LKO^* and *Fgfr2^FF^* livers under chow diet conditions. RNA‐seq profiling revealed highly overlapping global expression patterns between the genotypes, as evidenced by principal component analysis and sample‐to‐sample correlation heatmaps (Figure 5E,F). The differential expression analysis identified a limited number of differentially expressed genes between the genotypes (Figure S14A). Hierarchical clustering revealed highly similar global gene expression patterns between *Fgfr2^LKO^* and *Fgfr2^FF^* livers (Figure S14B). GO enrichment analysis did not show significant enrichment of fibrogenic, stress‐response or injury‐associated biological processes (Figure S14C). Although a small subset of differentially expressed genes was associated with extracellular matrix‐related terms, these genes did not demonstrate coordinated upregulation of canonical profibrotic markers and lacked a coherent fibrogenic signature (Figure S14D). Consistently, KEGG pathway analysis revealed enrichment across diverse signalling and metabolic pathways rather than pathways traditionally associated with fibrotic remodelling (Figure S14E,F). Collectively, these data suggest that the deletion of FGFR2 results in only minor adaptive transcriptional changes under normal conditions and does not predispose the cells to a profibrotic or injury‐sensitive state.

At the cellular level, FGFR2 expression was selectively inhibited in primary mouse hepatocytes using siRNA‐mediated knockdown. Among the three independent siRNA sequences evaluated, siRNA‐3 achieved substantial and consistent suppression of FGFR2 protein expression, as verified by western blot analysis (Figure S15). Collectively, these data confirm the effective hepatocyte‐specific deletion and siRNA‐mediated suppression of FGFR2, thereby facilitating subsequent investigation into FGFR2‐dependent signalling pathways and functional responses.

### 3.9 Hepatocyte FGFR2 mediates FGF10‐driven transcriptional remodelling during fibrosis

Building upon the genetic and molecular validation previously discussed, we investigated the necessity of hepatocyte FGFR2 for the therapeutic effects of FGF10 in the context of fibrotic injury. To eliminate confounding factors associated with AAV and to ensure consistent exposure across different genotypes, we administered rhFGF10 directly in a CCl_4_ model. Daily administration of rhFGF10 resulted in a dose‐dependent reduction of collagen deposition in the livers of mice injured with CCl_4_, achieving maximal efficacy at a dose of 5 mg/kg (Figure S16A–C). Furthermore, there was an inverse correlation between the administered dose and the SR‐positive area (Figure S16D).

Based on these findings, we compared the responses to CCl_4_ + rhFGF10 (5 mg/kg) between *Fgfr2^LKO^* and *Fgfr2^FF^* genotypes (Figure 5G). RNA‐seq revealed a significantly altered transcriptional response in the absence of hepatocyte FGFR2, with 1125 genes downregulated and 823 genes upregulated in *Fgfr2^LKO^* compared to *Fgfr2^FF^* (Figure 5H). GO enrichment analysis highlighted the involvement of extracellular space, ECM and collagen‐containing structures (Figure 5I), underscoring the role of hepatocyte FGFR2 as a critical mediator through which FGF10 modulates fibrotic gene networks.

### 3.10 RhFGF10 mitigates hepatic injury and fibrogenesis in an FGFR2‐dependent manner

**FIGURE 6:**
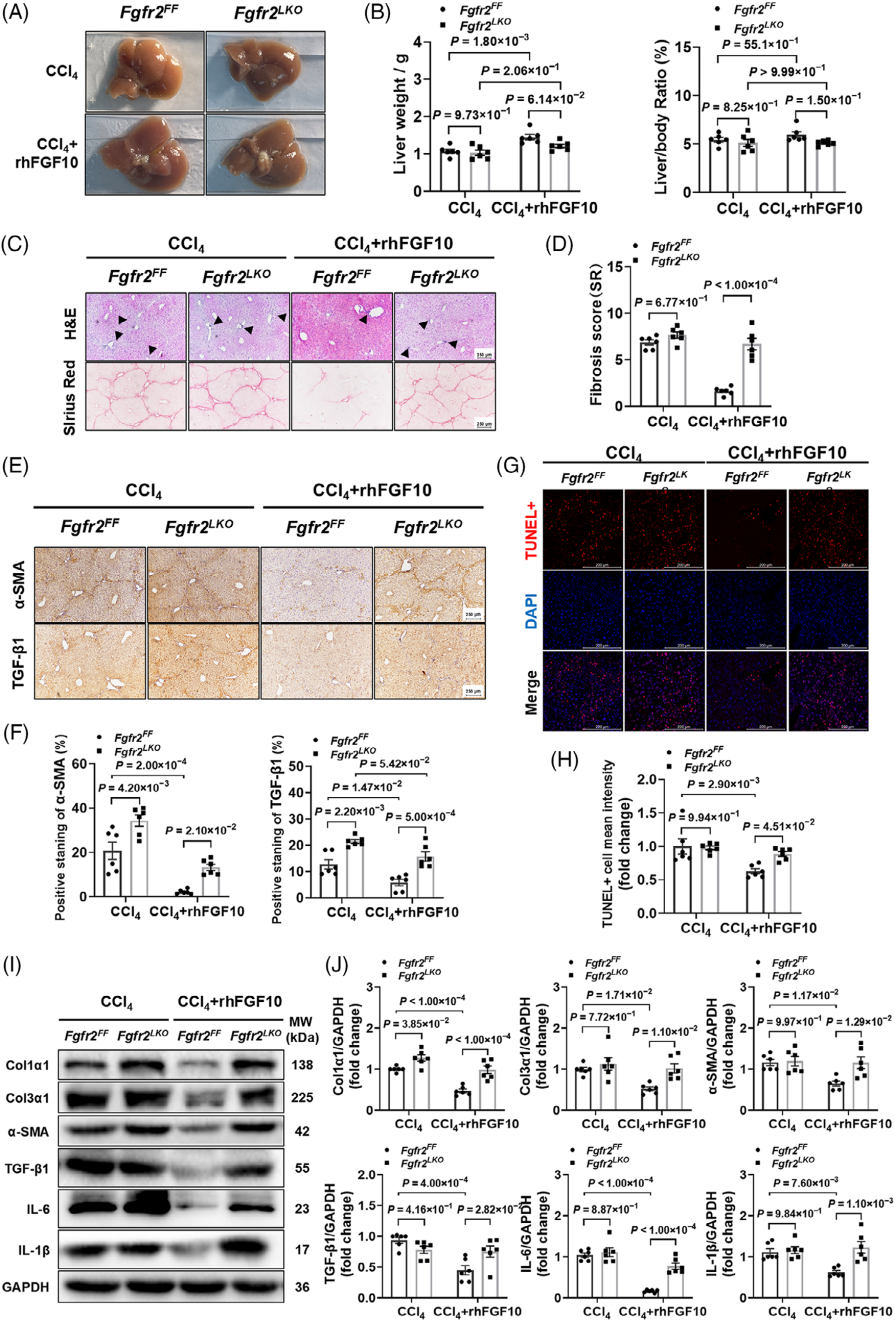
Hepatocyte fibroblast growth factor receptor 2 (FGFR2) is essential for mediating the antifibrotic, anti‐inflammatory and anti‐apoptotic effects of fibroblast growth factor 10 (FGF10) in CCl_4_‐injured mouse liver. (A) Overall liver morphology (*n* = 4 per group). (B) Liver weight and liver to body weight ratio (*n* = 6 per group). (C–D) haematoxylin and eosin (H&E), Sirius Red (SR) staining and SR and morphometric analysis. Scale bar, 250 µm. (E–F) Immunohistochemistry (IHC) and quantification of α‐smooth muscle actin (α‐SMA) and transforming growth factor β1 (TGF‐β1) expression (*n* = 6 per group). (G–H) Terminal deoxynucleotidyl transferase dUTP nick end labelling (TUNEL) assay images and quantification (*n* = 6 per group). Scale bar, 50 µm. (I–J) Immunoblot analysis and quantification of Col1α1, Col3α1, α‐SMA, TGF‐β1, IL‐6 and IL‐1β (*n* = 6 per group). Stats: two‐way analysis of variance (ANOVA) (B, F, H, J); unpaired *t*‐test (D).

To determine if the hepatoprotective effects of rhFGF10 necessitate the presence of hepatocyte FGFR2 during CCl_4_‐induced fibrosis, we conducted a series of analyses. At the study's conclusion, no significant differences in liver weight or liver‐to‐body‐weight ratio were observed between *Fgfr2^LKO^* and *Fgfr2^FF^* mice under rhFGF10 treatment (Figure 6A,B). Histological examination revealed that *Fgfr2^FF^* mice receiving rhFGF10 exhibited reduced hepatocyte injury, as evidenced by H&E staining, and decreased collagen deposition and fibrosis scores, as indicated by SR staining (Figure 6C,D). In contrast, these protective effects were absent in *Fgfr2^LKO^* mice (Figure 6C,D). Consistent with these observations, rhFGF10 treatment resulted in decreased expression of α‐SMA and TGF‐β1 in an FGFR2‐dependent manner in *Fgfr2^FF^* mice, but not in *Fgfr2^LKO^* mice (Figure 6E,F). Furthermore, TUNEL staining demonstrated a reduction in hepatic apoptosis in *Fgfr2^FF^* mice with rhFGF10, an effect not observed in *Fgfr2^LKO^* mice (Figure 6G,H). At the protein level, rhFGF10 administration led to a decrease in fibrosis markers (Col1α1, Col3α1, α‐SMA) and pro‐inflammatory cytokines (TGF‐β1, IL‐6, IL‐1β) in *Fgfr2^FF^* mice, whereas these suppressive effects were diminished or absent in *Fgfr2^LKO^* mice (Figure 6I,J). Collectively, these results indicate that the antifibrotic and anti‐inflammatory effects of rhFGF10 in vivo are contingent upon FGFR2.

### 3.11 Hepatocyte FGFR2 mediates FGF10‐driven FRS2α‐GSK3β activation and NF‐κB inhibition

**FIGURE 7:**
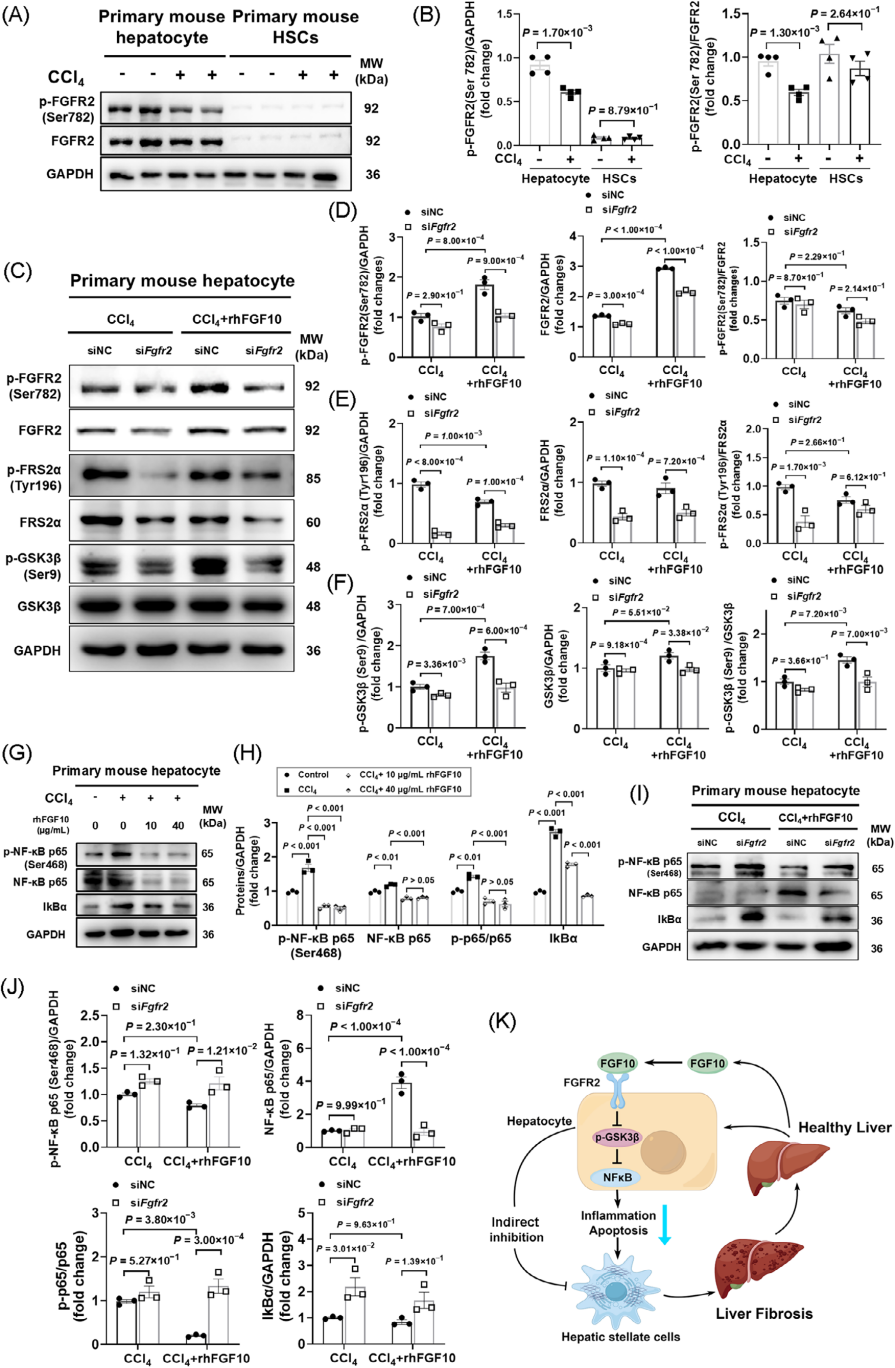
Hepatocyte fibroblast growth factor receptor 2 (FGFR2) signalling mediates the antifibrotic effects of fibroblast growth factor 10 (FGF10). (A) Immunoblot analysis was conducted to assess phosphorylated FGFR2 (p‐FGFR2, Ser782) and total FGFR2 levels in primary mouse hepatocytes and hepatic stellate cells (HSCs) subjected to treatment with or without CCl_4_ for a duration of 6 h. (B) The quantification of p‐FGFR2 was normalized to total FGFR2 in both hepatocytes and HSCs (*n* = 4 per group). (C) Representative immunoblots were obtained to illustrate the levels of phosphorylated and total FGFR2, FGFR substrate 2α (FRS2α) and glycogen synthase kinase 3β (GSK3β) in primary mouse hepatocytes transfected with either control siRNA (siNC) or Fgfr2 siRNA (*siFgfr2*), followed by treatment with CCl_4_ alone or in combination with recombinant human FGF10 (rhFGF10; 40 µg/mL) for 24 h (*n* = 3 per group). (D–F) Densitometric quantification was performed for the ratios of p‐FGFR2/FGFR2 (D), p‐FRS2α/FRS2α (E) and p‐GSK3β/GSK3β (F) corresponding to the data presented in (C). (G–H) Immunoblot analysis and subsequent quantification were carried out for IκBα, total nuclear factor kappa B (NF‐κB) p65 and phosphorylated NF‐κB p65 in primary mouse hepatocytes following a 24‐h exposure to CCl_4_, with or without the addition of rhFGF10 (*n* = 3 per group). (I–J) Immunoblot analysis and subsequent quantification were conducted to assess the levels of IκBα, total NF‐κB p65 and phosphorylated NF‐κB p65 in primary mouse hepatocytes following the knockdown of Fgfr2. This was followed by treatment with either CCl_4_ alone or a combination of CCl_4_ and recombinant human FGF10 for a duration of 24 h (*n* = 3 per group). (K) A schematic representation illustrates that FGF10 activation of FGFR2 signalling in hepatocytes results in the activation of the FRS2α‐GSK3β pathway, which suppresses NF‐κB signalling. This suppression leads to a reduction in the secretion of pro‐inflammatory cytokines (TNF‐α, transforming growth factor β1 [TGF‐β1] and IL‐1β) by hepatocytes, thereby indirectly mitigating the activation of hepatic stellate cells and the deposition of extracellular matrix during CCl_4_‐induced liver fibrosis. Data are presented as mean ± SEM. Statistical significance was assessed using a two‐tailed unpaired Student's *t*‐test (B) and ordinary two‐way analysis of variance (ANOVA) (D‐F, H, J).

To elucidate the downstream mechanisms, we investigated key effectors in primary cells following CCl_4_ exposure. In primary mouse hepatocytes, CCl_4_ exposure resulted in a reduction of phosphorylation of FGFR2 at Ser782, with minimal alterations observed in HSCs (Figure 7A,B), suggesting a hepatocyte‐centric effect. Treatment with rhFGF10 restored FGFR2 phosphorylation in CCl_4_‐treated hepatocytes to levels comparable to the control group (siNC) (Figure 7C,D). Knockdown of Fgfr2 (si*Fgfr2*) attenuated the rhFGF10‐induced phosphorylation of FRS2α at Tyr196 and GSK3β at Ser9 (Figure 7C,E,F), thereby identifying FRS2α and GSK3β as FGFR2‐dependent mediators of FGF10 signalling in injured hepatocytes. Given that inflammatory signalling contributes to fibrogenesis, we evaluated NF‐κB activity. RhFGF10 was found to reduce p65 phosphorylation and stabilize IκBα (Figure 7G,H); however, these anti‐inflammatory effects were significantly diminished following FGFR2 knockdown in hepatocytes (Figure 7I,J). Collectively, these findings suggest that hepatocyte FGFR2 acts upstream of FRS2α‐GSK3β activation and NF‐κB inhibition in the FGF10 response (Figure 7K).

## 4 DISCUSSION

This study elucidates a hepatocyte‐centric FGF10‐FGFR2 signalling pathway that mitigates inflammatory responses, maintains hepatocyte viability, and both limits and partially remodels fibrotic architecture. The expression of hepatic FGF10 and FGFR2 proteins undergo dynamic alterations throughout disease progression, characterized by an initial transient induction of FGF10 protein during acute injury in murine models, followed by an overall decline at advanced fibrotic stages in both human patients and mouse models. Augmentation of FGF10 levels, achieved by liver‐targeted gene delivery or administration of recombinant protein, was associated with reduced hepatocyte apoptosis, diminished inflammation and decreased ECM deposition. Conversely, the hepatocyte‐specific deletion of FGFR2 negated these beneficial effects, thereby establishing FGFR2 as the essential receptor for FGF10‐mediated protection in the context of chronic injury.

Notably, FGF10 not only decelerated disease progression, but also induced histological regression compared to time‐matched controls, thereby elevating the FGF10–FGFR2 axis from a mere protective mechanism to a hepatocyte‐centred reversal strategy. These findings suggest that the loss of FGFR2 in hepatocytes represents a critical signalling bottleneck, potentially exacerbated by the spatial restriction of paracrine FGF10 within the fibrotic microenvironment. In alignment with recent high‐resolution spatial analyses indicating that short‐range signalling in advanced fibrosis increasingly relies on close cellular proximity,^29^ paracrine FGF10 signalling to hepatocytes is likely to be particularly susceptible to both receptor loss and diminished spatial ligand accessibility at the disease progression. FGF10 is a well‐characterized paracrine ligand that preferentially interacts with the epithelial FGFR2b isoform.^32^, ^33^, ^34^ In this study, discrimination between FGFR2 isoforms was not conducted, particularly at the protein level, due to the absence of isoform‐selective antibodies. Consequently, although we did not directly determine FGFR2 isoform utilization, the hepatocyte‐dependent effects observed are most consistent with canonical epithelial FGFR2 signalling. Future spatially resolved methodologies, such as RNAscope‐based analyses, will further enhance the precision of ligand‐receptor mapping in fibrotic liver tissue.^35^

Mechanistically, FGF10 engages FGFR2/FRS2α signalling pathways in hepatocytes, leading to an increase in GSK3β (Ser9) inhibitory phosphorylation and a subsequent suppression of NF‐κB activity. This signalling cascade is instrumental in maintaining epithelial integrity, reducing cytokine production, including TGF‐β1, and indirectly limiting HSC activation and collagen synthesis. These findings are consistent with the established roles of GSK3β and NF‐κB in hepatocyte stress responses and the amplification of inflammatory processes, as documented in studies.^36^, ^37^, ^38^, ^39^, ^40^ They highlight the GSK3β‐NF‐κB axis as a pivotal effector through which FGF10 confers hepatoprotective effects and exerts primary control over fibrogenic signalling in hepatocytes. The relatively elevated concentrations of rhFGF10 employed in vitro were chosen to ensure robust activation of FGFR2‐dependent signalling pathways under conditions of acute injury. Unlike in vivo environments, cell culture systems lack stromal sequestration, extracellular matrix binding and spatial ligand gradients that are crucial for modulating local FGF10 availability. Therefore, these concentrations were utilized to explore signalling mechanisms rather than to directly replicate physiological ligand levels.

These findings elucidate the specific roles within hepatic FGF signalling pathways. Endocrine FGFs, such as FGF19 and FGF21, interact with FGFR4/FGFR1c in conjunction with β‐Klotho to regulate bile acid and metabolic homeostasis, and they are currently being explored in clinical trials for MASLD.^41^, ^42^, ^43^ In contrast, paracrine FGFR2b ligands, including FGF7 and FGF10, have primarily been investigated in the context of epithelial regeneration,^44^, ^45^ with their roles in fibrogenesis being less well‐defined. The demonstration that hepatocyte FGFR2 is essential for the antifibrotic effects of FGF10 highlights an intrinsic epithelial function of the FGFR2 pathway in chronic liver disease, complementing the metabolic functions associated with FGFR4.^19^, ^46^ Our findings further suggest that targeted enhancement of FGFR2 in response to FGF10 administration corresponds with a reduction in profibrotic transcriptional activity, supporting a receptor‐centred mechanism wherein hepatocytes serve as key regulators of the fibrotic microenvironment.

The temporal and compartment‐specific regulation of FGF10 presents an additional layer of complexity. In cases of acute or short‐term toxicant injury, as demonstrated in previous studies and our preliminary CCl_4_ time‐course experiments, there is a transient induction of hepatic FGF10, indicative of a compensatory pro‐regenerative response. However, with prolonged CCl_4_ exposure leading to advanced fibrosis, whole‐liver FGF10 expression exhibits a biphasic pattern and eventually decline. Notably, the apparent discrepancy between increased transcriptional activity in individual HSCs and the declining overall hepatic FGF10 levels is attributed to alterations in cellular composition and spatial distribution, rather than a sustained increase in tissue‐level ligand availability. As fibrosis advances, the reduction in the number and spatial accessibility of FGF10‐expressing cells constrains the effective hepatic pool of FGF10, despite localized transcriptional activity within activated HSCs. Alongside the concurrent decrease in hepatocyte FGFR2 expression, these findings support a model wherein the disruption of the FGF10–FGFR2 paracrine axis is associated with the progression of fibrosis, thereby contributing to the diminished epithelial protective signalling observed in advanced stages of the disease.

A significant translational insight is that histological improvement may precede biochemical normalization. In the context of CCl_4_‐induced injury, FGF10 was observed to reduce bridging fibrosis, α‐SMA expression and inflammatory signalling despite minimal alterations in serum aminotransferase levels. This phenomenon aligns with the concepts of hepatic recompensation and injury tolerance, where structural repair and matrix remodelling can occur under conditions of sustained or repeated toxic injury prior to the complete normalization of biochemical markers. This has been documented in recent experimental models of repeated hepatotoxic exposure and adaptive liver remodelling.^47^, ^48^, ^49^ Alongside findings from acute injury models,^31^ these data suggest that FGF10 facilitates a conserved pro‐survival and pro‐regenerative response while mitigating inflammation. Consequently, the observed reduction in collagen deposition and simplification of fibrotic architecture, when compared to time‐matched controls, should be interpreted as indicative of partial regression‐associated remodelling rather than a definitive reversal of fully established fibrosis.

Of significant translational relevance, it is noteworthy that while our study lacks a delayed “post‐establishment” treatment arm, rhFGF10 administration commenced after 2 weeks of CCl_4_ exposure. At this juncture, the mice had already developed pronounced bridging fibrosis, and CCl_4_ exposure was maintained. Under these experimental conditions, FGF10 not only halted further fibrotic progression but also eradicated bridging fibrosis, as evidenced by SR staining. This finding indicates histologic regression‐associated remodelling compared to time‐matched controls, rather than merely suppressing progression. Consequently, this underscores the therapeutic potential of the hepatocyte‐FGFR2 axis, even amidst ongoing injury.

In the context of metabolic toxicity induced by HFD combined with CCl_4_ exposure, FGF10 demonstrated sustained antifibrotic efficacy, which was associated with selective metabolic remodelling, including the downregulation of *Fasn* and *Srebf2*. Transcriptomic analysis revealed enrichment of lipid and fatty acid pathways following treatment with AAV‐*Fgf10* or rhFGF10. This supports a model in which epithelial protection is linked to targeted metabolic remodelling (see Figure S17), thereby mitigating the inflammatory and profibrogenic burden in steatohepatitis. These findings underscore the preserved antifibrotic efficacy under conditions of metabolic priming and provide evidence for regression beyond a purely toxicant‐induced context. It further suggests that hepatocyte‐centred reversal mechanisms can be sustained even in the presence of complex metabolic disease.

These findings underscore the role of hepatocytes as pivotal upstream regulators within the fibrotic niche.^50^, ^51^ This prioritization is corroborated by genetic evidence demonstrating that the hepatocyte‐specific deletion of FGFR2 negates the antifibrotic effects of FGF10 in vivo, thereby establishing hepatocytes as the principal functional responders in this context. Instead of solely targeting HSCs, the FGF10–FGFR2‐mediated modulation of hepatocyte secretory activity diminishes levels of TGF‐β1 and pro‐inflammatory cytokines, thereby exerting a paracrine inhibitory effect on HSC activation.^52^, ^53^, ^54^ This hepatocyte‐centric approach aligns with emerging paradigms concerning HSC plasticity, immune‐metabolic interactions and epithelial stress responses as co‐contributors to fibrosis.^55^, ^56^ It is important to acknowledge that other cell types, such as Kupffer cells, cholangiocytes and endothelial cells, may also play a role in the response. Future ex vivo studies that pair hepatocytes derived from injured livers with other types of cells, alongside receptor perturbation in hepatocytes, will be instrumental in elucidating how FGFR2‐intrinsic signals remodel the fibrotic microenvironment. This baseline transcriptional shift resulting from the deletion of FGFR2 under chow diet conditions in mice likely represents adaptive remodelling rather than a pathological state and does not inherently confer a predisposition to fibrogenesis.

A critical translational consideration pertains to the proliferative potential associated with FGFR2 signalling. Activation of FGFR2 has been implicated in epithelial cell proliferation and tumorigenesis in specific organs and disease contexts, most notably in gastric cancer and cholangiocarcinoma.^57^, ^58^, ^59^ This raises theoretical concerns that sustained or uncontrolled FGF10 signalling could pose an oncogenic risk. However, in the present study, neither AAV‐mediated hepatic expression of FGF10 nor the administration of recombinant FGF10, at the evaluated doses and treatment durations, resulted in hepatomegaly, aberrant tissue growth or tumour‐like abnormalities. Instead, the predominant effects of FGF10 were a reduction in hepatocyte apoptosis and a decrease in inflammatory signalling, aligning with a regenerative and cytoprotective role rather than the induction of pathological proliferation. Nonetheless, long‐term studies examining dose responsiveness, extended treatment regimens and potential oncogenicity are crucial for the progression of FGF10–FGFR2‐targeted therapies toward clinical application.

It is important to acknowledge the limitations of this study. CCl_4_ primarily serves as a model for toxicant‐induced fibrosis characterized by bridging septa and does not fully replicate the characteristics of cholestatic, immune‐dominant or purely lipotoxic diseases. To enhance external validity, we incorporated dietary models and human tissues into our research; however, further formal testing in lipotoxic and immune‐biased contexts is necessary. Considering the expression of FGFR2 in cholangiocytes and progenitor cell compartments, a more detailed examination of the epithelial‐stromal interface is warranted. This can be achieved through the application of single‐cell transcriptomics and lineage tracing techniques. The inflammatory changes delineated in this study primarily reflect hepatocyte‐intrinsic regulation of cytokine output and tissue‐level inflammatory signalling, rather than a direct examination of immune cell behaviour. Mechanisms related to the recruitment or activation of immune cells were not directly evaluated and warrant investigation in future studies employing specific immunophenotyping methodologies. For translational purposes, a comprehensive understanding of pharmacokinetics, receptor selectivity and long‐term safety is essential, particularly in light of the proliferative potential associated with FGFR2‐driven epithelial pathways. A focused study that initiates FGF10 treatment following the establishment of advanced fibrosis, utilizing time‐matched comparators, will further elucidate reversal kinetics and assess whether reinforcement of epithelial FGFR2 can lead to clinically significant regression.

In summary, chronic injury leads to the downregulation of FGF10, which consequently weakens FGFR2–FRS2α signalling in hepatocytes, reduces the inhibition of GSK3β and enhances NF‐κB activity. This pro‐inflammatory environment exacerbates hepatocyte loss and stimulates HSC activation and ECM deposition. Restoration of FGF10/FGFR2 signalling through recombinant protein administration, vector‐based delivery or selective FGFR2 agonists disrupts this pathological cascade, thereby preserving hepatocyte integrity and mitigating fibrosis. This pathway not only complements endocrine FGF therapies but also underscores the importance of epithelial repair and inflammation control at the site of hepatocyte injury. The necessity of hepatocyte FGFR2, along with observable histologic regression, provides robust mechanistic and phenotypic evidence supporting a hepatocyte‐focused strategy for reversing fibrosis.

## 5 CONCLUSION

In conclusion, our study elucidates an intrinsic FGF10–FGFR2 signalling axis that plays a pivotal role in constraining liver fibrogenesis. Through investigations in both human and murine models, we observe a decline in hepatic FGF10 levels with advancing disease stages. Notably, the augmentation of FGF10 results in a reduction of collagen deposition, α‐SMA expression and inflammation, even in metabolically primed liver disease. These therapeutic effects are contingent upon the presence of hepatocyte FGFR2. Mechanistically, FGF10 facilitates the restoration of FGFR2 phosphorylation, activates FRS2α, enhances GSK3β (Ser9) phosphorylation and suppresses NF‐κB activity, thereby mitigating inflammatory injury and apoptosis while indirectly inhibiting HSC activation. Collectively, these findings advocate for the hepatocyte‐targeted enhancement of FGFR2 signalling as a viable translational approach. This strategy, whether employed as a monotherapy or in conjunction with metabolic agents, holds promise for reprogramming the fibrotic niche and promoting regression‐associated fibrotic remodelling in cases of advanced liver fibrosis.

## AUTHOR CONTRIBUTIONS

*Conceptualization*: Xuanxin Yang, Xiaojie Wang and Xiaokun Li. *Formal analysis*: Bingjie Yu, Huan Wang and Yuandong Xu. *Funding acquisition*: Xiaojie Wang, Xuanxin Yang and Xiaokun Li. *Investigation*: Xuanxin Yang, Qingqing Dong, Jiaying Ma, Yawei Yan, Shuangyan Peng, Qiqi Wu, Tingting Zhang, Panyu Zhang, Zelong Jiang, Chao Lu, Le Li, Xinyi You and Zhixiang Mu. *Methodology*: Xuanxin Yang, Qingqing Dong, Jiaying Ma and Yawei Yan. *Project administration*: Xuanxin Yang, Xiaojie Wang and Bingjie Yu. *Resources*: Yawei Yan, Bingjie Yu, Qi Hui, Minghua Zheng and Zhixiang Mu. *Supervision*: Xiaojie Wang, Xiaokun Li and Minghua Zheng. *Validation*: Xuanxin Yang, Qingqing Dong, Jiaying Ma, Yawei Yan and Bingjie Yu. *Visualization*: Xuanxin Yang, Jiaying Ma, Yawei Yan and Yuandong Xu. *Writing—original draft*: Xuanxin Yang and Xiaojie Wang. *Writing—review and editing*: Xuanxin Yang, Xiaojie Wang, Bingjie Yu, Huan Wang, Joshua Banda and Qi Hui.

## CONFLICT OF INTEREST STATEMENT

The authors declare no conflicts of interest.

## ETHICAL APPROVAL

All procedures involving human participants were approved by the Ethics Committee of Clinical Research at the First Affiliated Hospital of Wenzhou Medical University (approval no. 2016/246), and written informed consent was obtained from all participants. No samples were obtained from executed prisoners or institutionalized individuals. All animal experiments were approved by the Animal Ethics Committee of Wenzhou Medical University (approval no. wydw2022‐0691) and were conducted in accordance with institutional guidelines for the care and use of laboratory animals.

## Supporting information



Supporting Information

Supporting Information

## Data Availability

The data that support the findings of this study are available from the corresponding author upon reasonable request. Raw and processed RNA‐seq data will be deposited in GEO upon acceptance. Additional datasets referenced in this study are available in the GEO database under accession numbers GSE246221 and GSE212837.
