# Supplementary material for: Fibroblast growth factor 10 attenuates advanced liver fibrosis through hepatocyte fibroblast growth factor receptor 2 signalling
Source: Clin Transl Med. 2026 May 12;16(5):e70675. doi: 10.1002/ctm2.70675 (PMC13162133; doi:10.1002/ctm2.70675)
Supplement: Supplementary file 1 — Supporting Information [file CTM2-16-e70675-s001.docx]

**SUPPLEMENTARY FILE**

**Supplementary Figures**

**Figure S1**

**
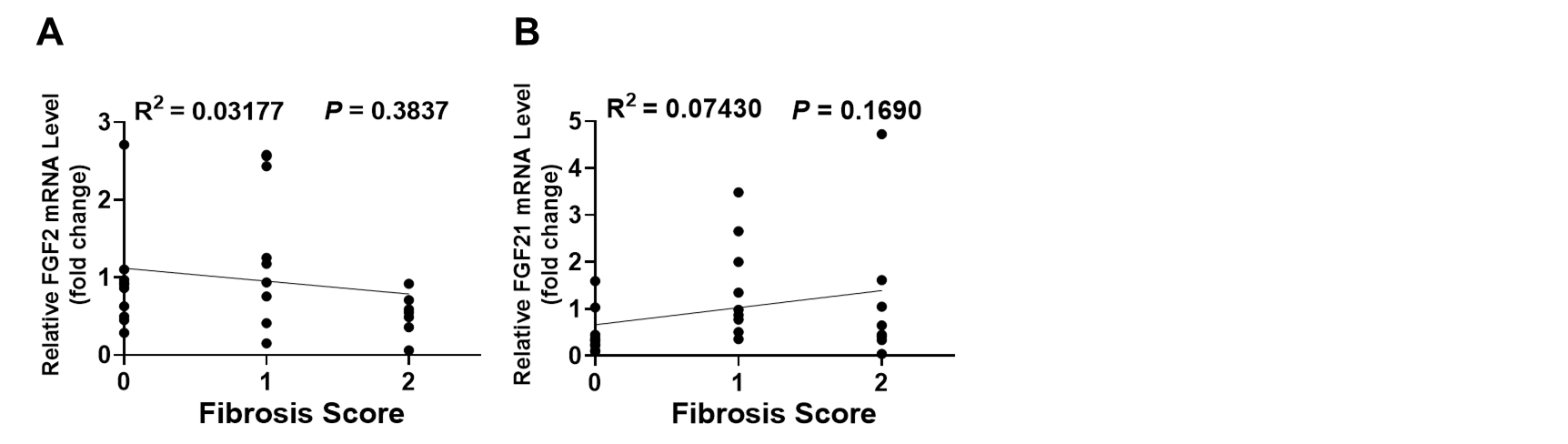
**

**Figure S1. The mRNA expression levels of hepatic *FGF2* and *FGF21* do not exhibit an association with the severity of fibrosis in MASLD.** (A-B) Quantitative PCR analysis of MASLD biopsy samples, categorized by fibrosis stage (FS), reveals no significant correlation between the expression of *FGF2* or *FGF21* and fibrosis stage. Linear regression: *FGF2,* R^2^=0.0318, P=0.384; *FGF21,* R^2^=0.0743, P=0.169. One-way ANOVA across FS0-FS2.

**Figure S2**

**
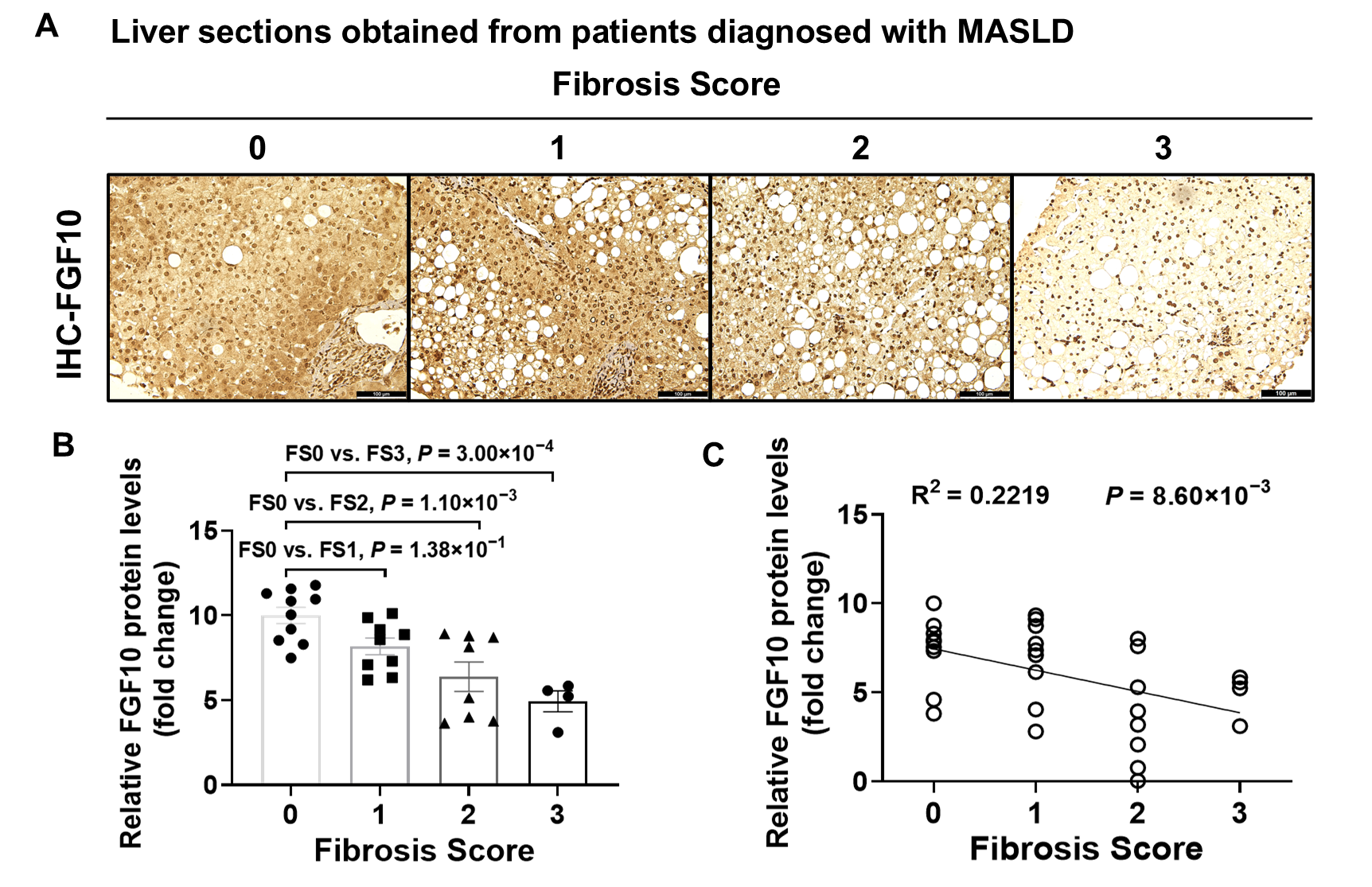
**

**Figure S2. The expression of FGF10 protein diminishes as fibrosis progresses.** (A) Immunohistochemistry (IHC) analysis across fibrosis stages FS0 to FS3 reveals a progressive reduction in FGF10 signal, with the lowest expression observed at FS3. Scale bars, 100 µm. (B-C) IHC analysis of FGF10 across stages FS0 to FS3, including quantitative assessment (B) and regression analysis against fibrosis stage (C). Stats: one-way ANOVA (B).

**Figure S3**


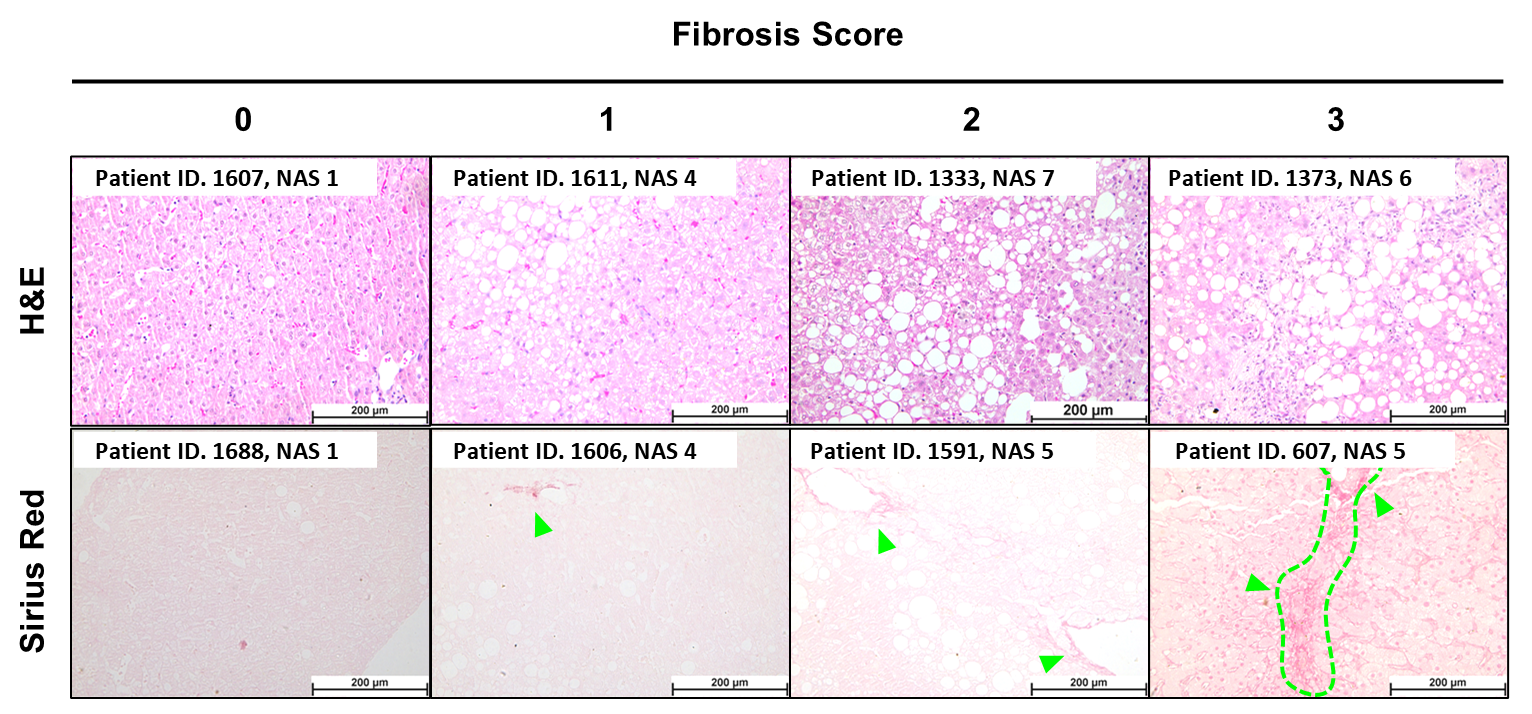


**Figure S3. Pathology reference panels for fibrosis stages FS0-FS3 are presented, with representative biopsies annotated according to MASH CRN staging and extracellular matrix (ECM) highlighted.** Scale bars, 200 µm.

**Figure S4**

**
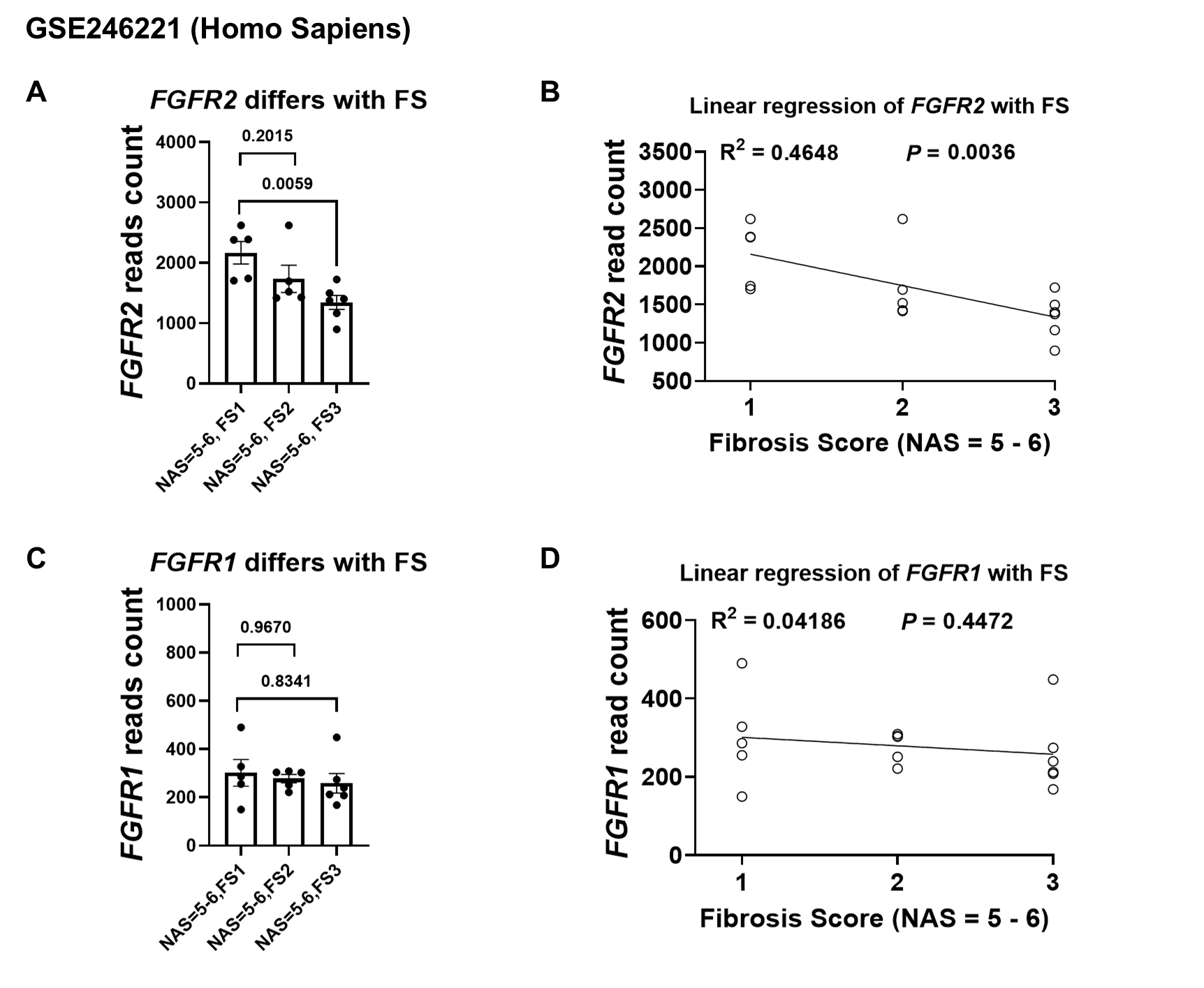
**

**Figure S4. External RNA-seq validation in human MASH using a publicly available bulk RNA-seq dataset (GSE246221).** This dataset includes 16 MASH patients with matched disease activity (NAS = 5-6) and demonstrates that *FGFR2*, rather than *FGFR1*, is inversely associated with fibrosis. (A) Hepatic *FGFR2* read counts across fibrosis stages FS1-FS3 for reference, with bars representing the mean ± SEM and dots indicating individual samples. (B) A linear regression analysis of *FGFR2* read counts versus fibrosis stages, revealing a significant negative association. (C) Hepatic *FGFR1* read counts across FS1-FS3 at NAS = 5-6. (D) A linear regression analysis of *FGFR1* read counts versus fibrosis stages, indicating no significant association. The data were reanalyzed from GSE246221, with samples restricted to NAS = 5-6 to maintain constant disease activity. The findings suggest that *FGFR2* transcript levels decrease with fibrosis progression, whereas *FGFR1* levels remain unchanged.

**Figure S5**

**
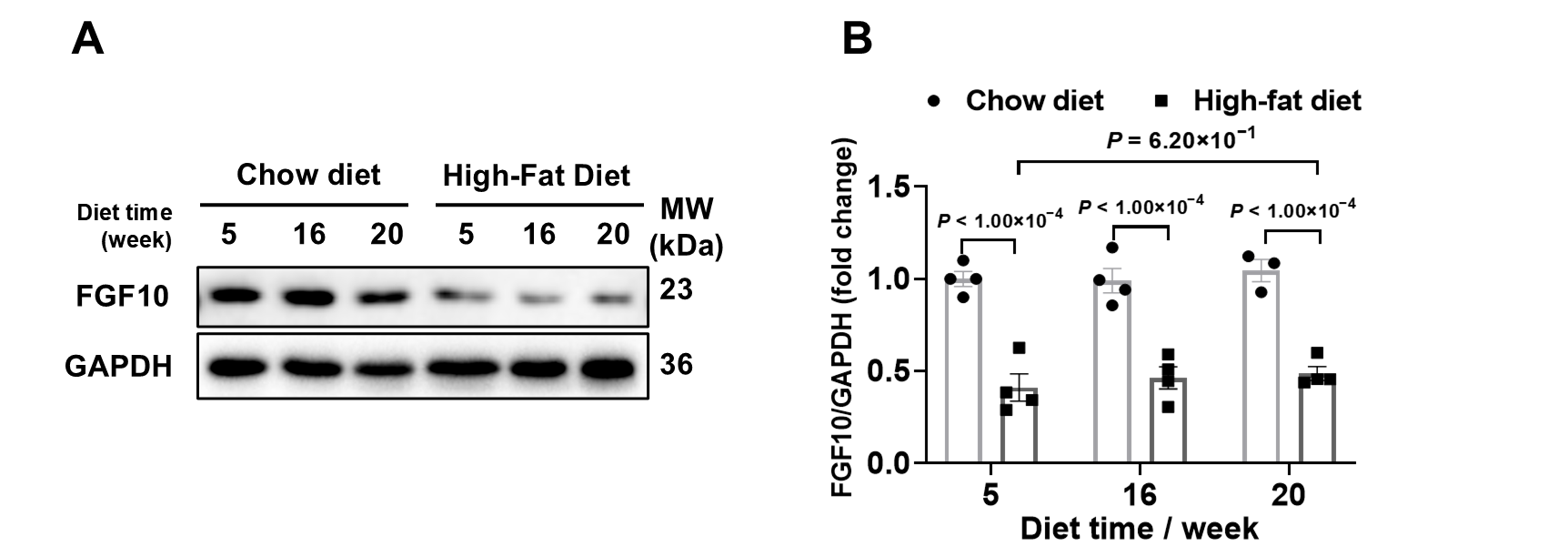
**

**Figure S5. A high-fat diet (HFD) leads to a modest reduction in hepatic FGF10 levels, with no evident time-dependent pattern.** (A) Western blot analysis of hepatic FGF10 protein in mice fed a chow diet compared to those on an HFD over a specified time course. (B) Quantitative analysis of Western blot results at 0, 5, 16, and 20 weeks for HFD versus chow-fed mice (n = 4). Statistical analysis was performed using two-way ANOVA (B).

**Figure S6**


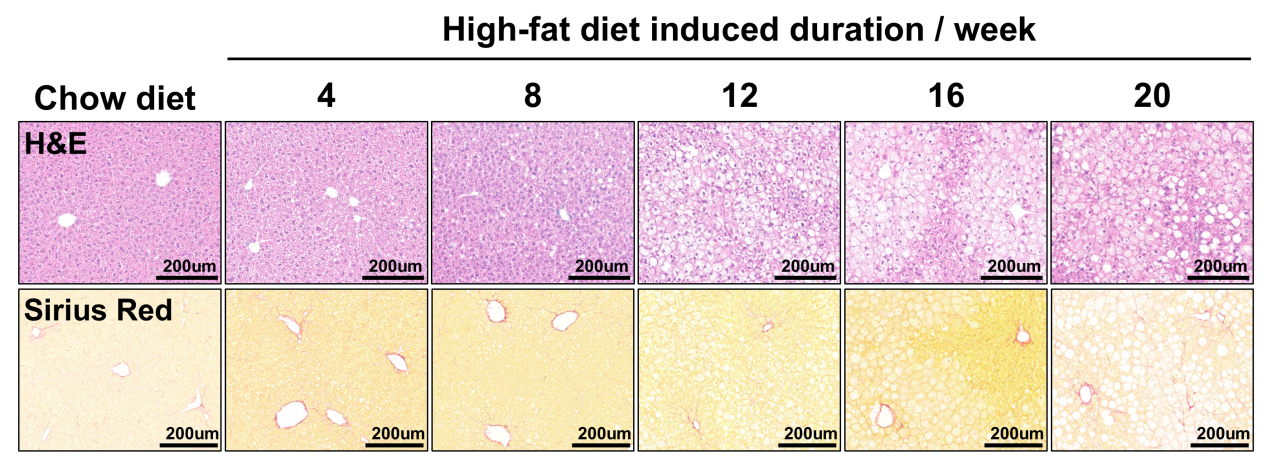


**Figure S6. High-fat diet (HFD) in isolation does not induce bridging fibrosis.** Hematoxylin and eosin (H&E) and Sirius Red staining over a period of 0-20 weeks reveal the presence of steatosis without the formation of septa or bridging fibrosis. Scale bars, 200 µm.

**Figure S7**

**
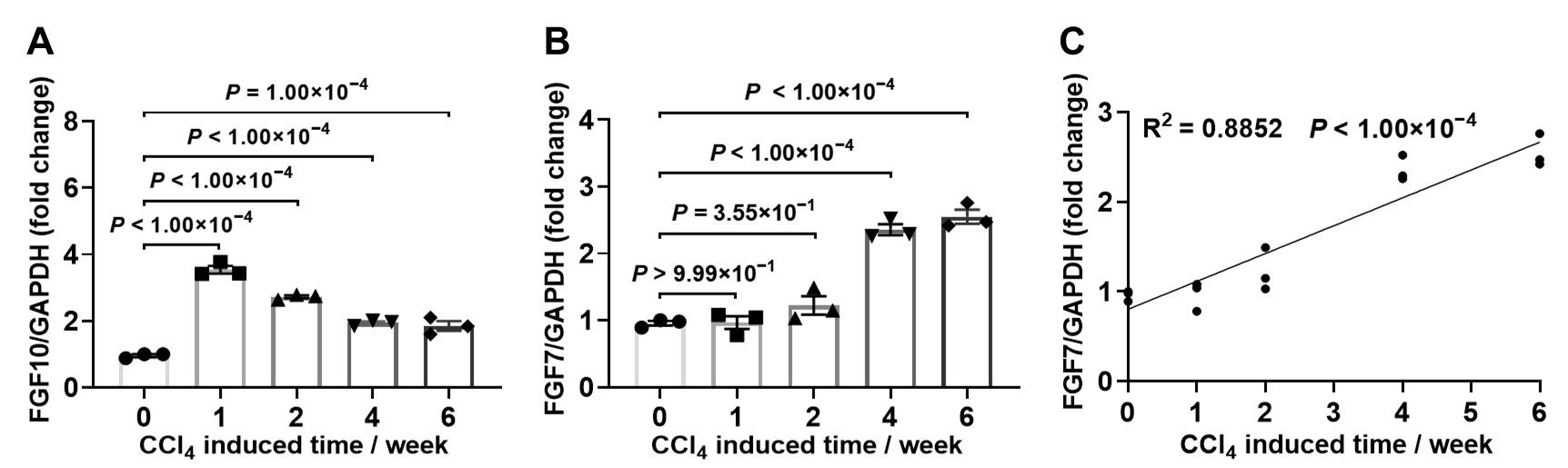
**

**Figure S7. Quantitative analysis of hepatic FGF10 and FGF7 expression during CCl_4_ exposure.** (A-B) Quantitative analysis of hepatic FGF10 (A) and FGF7 (B) protein levels was conducted using Western blotting at 0, 1, 2, 4, and 6 weeks following CCl_4_ treatment. Protein expression levels were normalized to GAPDH and presented as fold changes relative to the baseline measurement at week 0 (n = 3). (C) Linear regression analysis demonstrated a positive correlation between FGF7 expression and the duration of CCl_4_ exposure (R^2^ = 0.885, *P* < 1 × 10^-4^). Stats: one-way ANOVA (A, B).

**Figure S8**

**
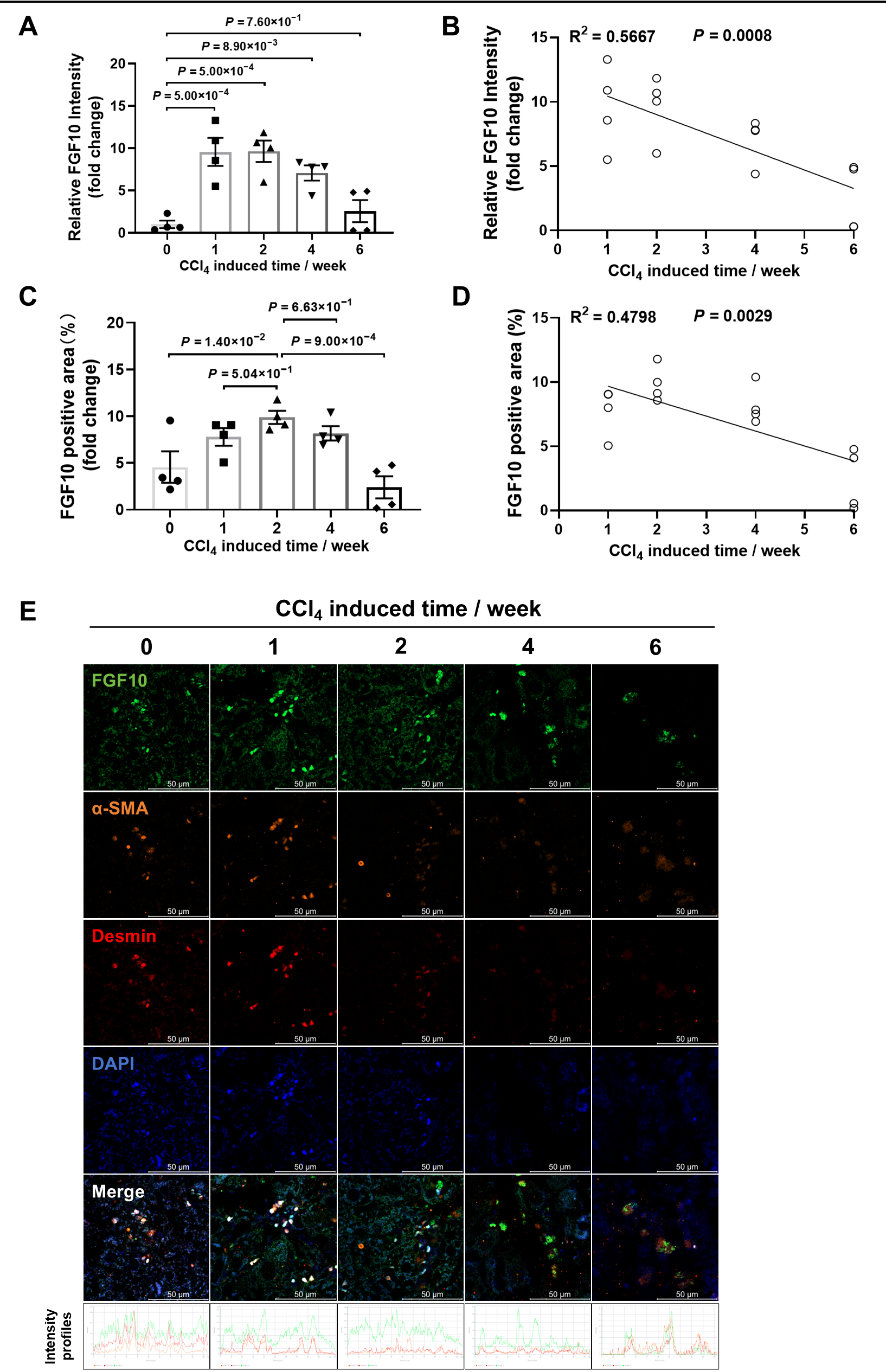
**

**Figure S8. Immunofluorescence-based analysis of hepatic FGF10 expression during CCl_4_-induced fibrosis.** (A) The relative intensity of FGF10 immunofluorescence was measured across the CCl_4_ treatment time course (n = 4). (B) A linear regression analysis was conducted to examine the relationship between relative FGF10 immunofluorescence intensity and the duration of CCl_4_ treatment (n = 4). (C) The FGF10-positive area was quantified as a percentage of the total field area at specified time points (n = 4). (D) A linear regression analysis was performed to assess the correlation between the FGF10-positive area and the duration of CCl_4_ treatment (n = 4). (E) High-magnification images from Figure 1L illustrate triple immunofluorescence staining for FGF10, α-SMA, and desmin, alongside DAPI staining. These images reveal the co-localization of FGF10 with hepatic stellate cell markers and demonstrate minimal nuclear signal, indicating a predominant cytoplasmic and pericellular localization of FGF10 in activated stellate cells. Stats: one-way ANOVA (A, C).

**Figure S9**

**
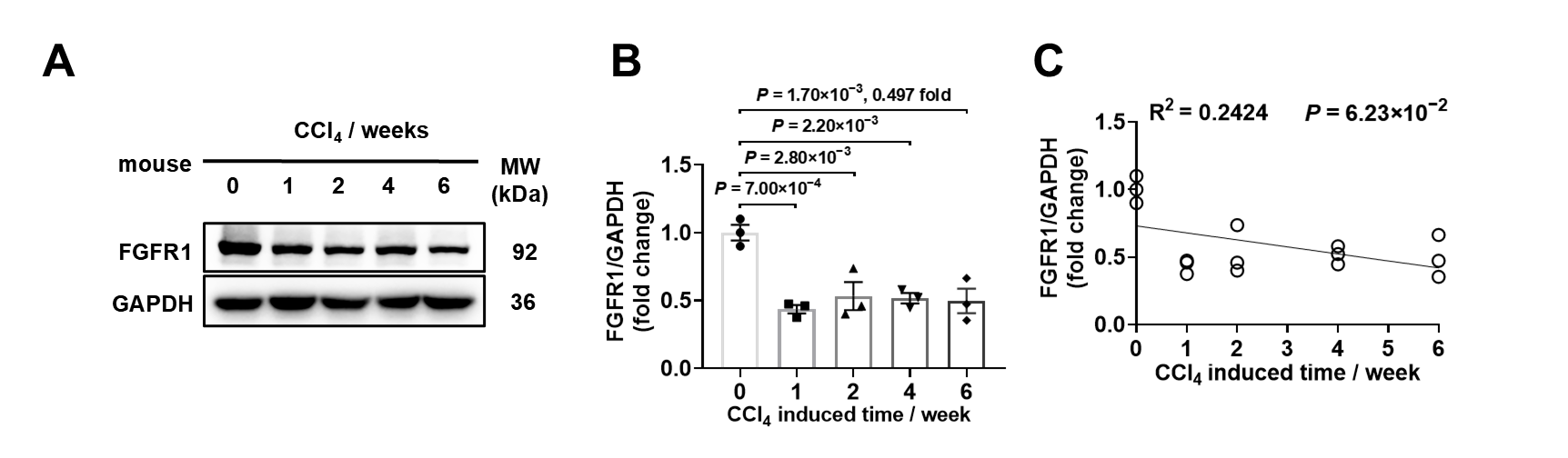
**

**Figure S9. FGFR1 changes modestly and plateaus during CCl_4_ exposure.** (A-C) Temporal analysis of hepatic FGFR1 expression following CCl_4_ exposure (A); quantitative assessment (B, n = 3) and regression analyses (C, n = 3). Stats: one-way ANOVA (B).

**Figure S10**

**
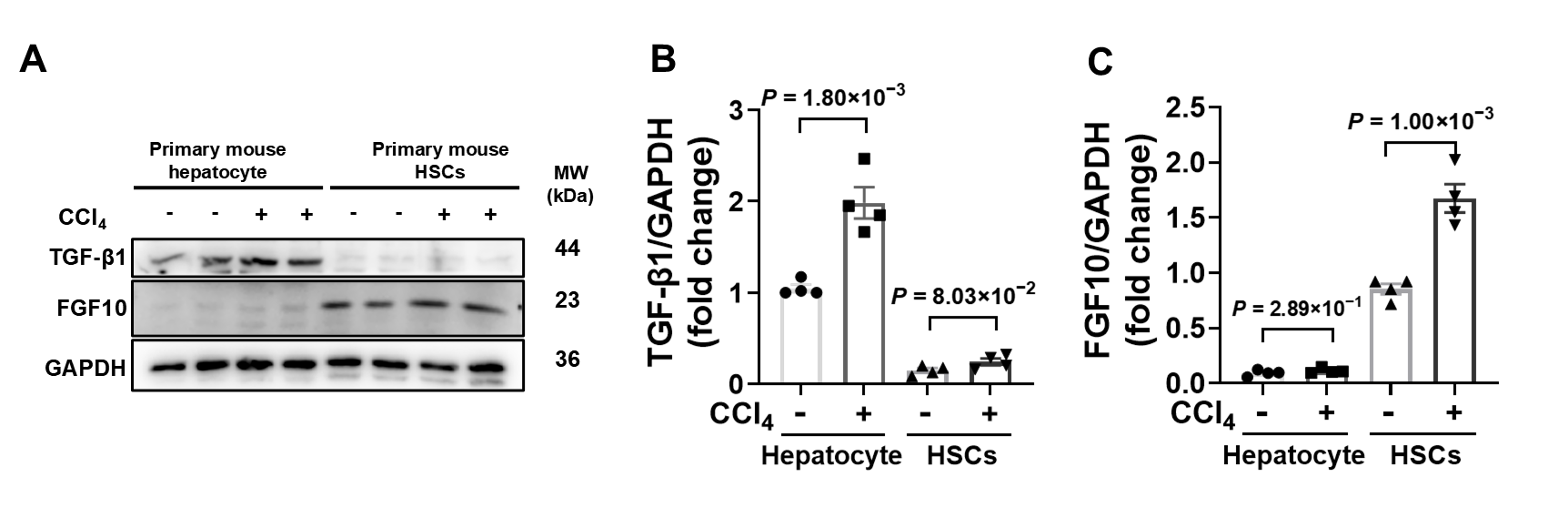
**

**Figure S10. Acute CCl_4_ differentially regulates hepatocytes vs HSCs *in vitro*.** (A-C) In primary hepatocytes, acute CCl_4_ exposure increases TGF-β1 expression, whereas in HSCs it induces a robust upregulation of FGF10 (n = 4). Stats: unpaired Student’s t-test (B, C).

**Figure S11**

**
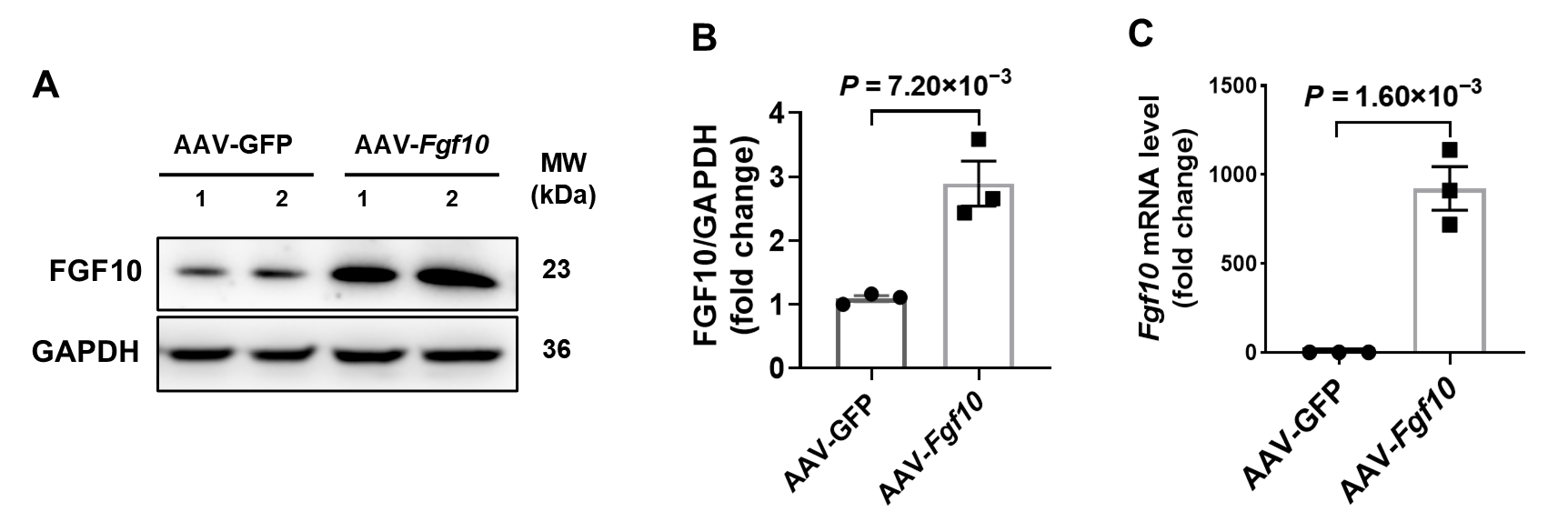
**

**Figure S11. AAV-*Fgf10* increases hepatic FGF10.** (A-C) Western blotting (WB) and quantitative Reverse Transcription Polymerase Chain Reaction (qRT-PCR) analyses confirm the induction of protein (A) and mRNA levels (B) compared to the adeno-associated virus expressing green fluorescent protein (AAV-GFP) control group (n = 3). Stats: unpaired t-test (B, C).

**Figure S12

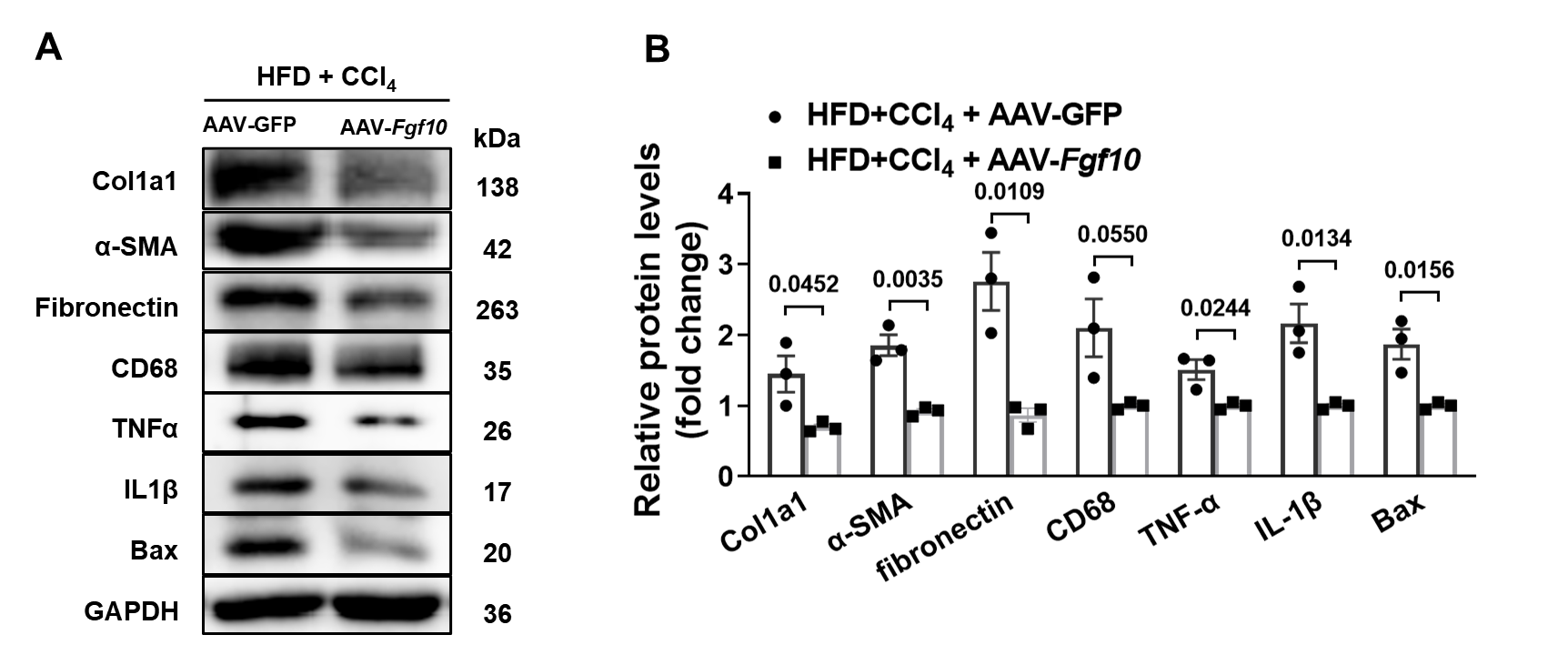
**

**Figure S12. Liver-specific FGF10 attenuates profibrotic, inflammatory, and apoptotic pathways in steatohepatitis induced by a high-fat diet and CCl_4_.** (A-B) Immunoblot analysis (A) demonstrates decreased expression levels of Col1a1, α-SMA, fibronectin, CD68, TNF-α, IL-1β, and Bax, using GAPDH as a loading control. Quantification (B) is normalized to GAPDH (n = 3). Statistics: data are mean ± SEM, with tests as indicated (unpaired t-test where shown).

**Figure S13**

**
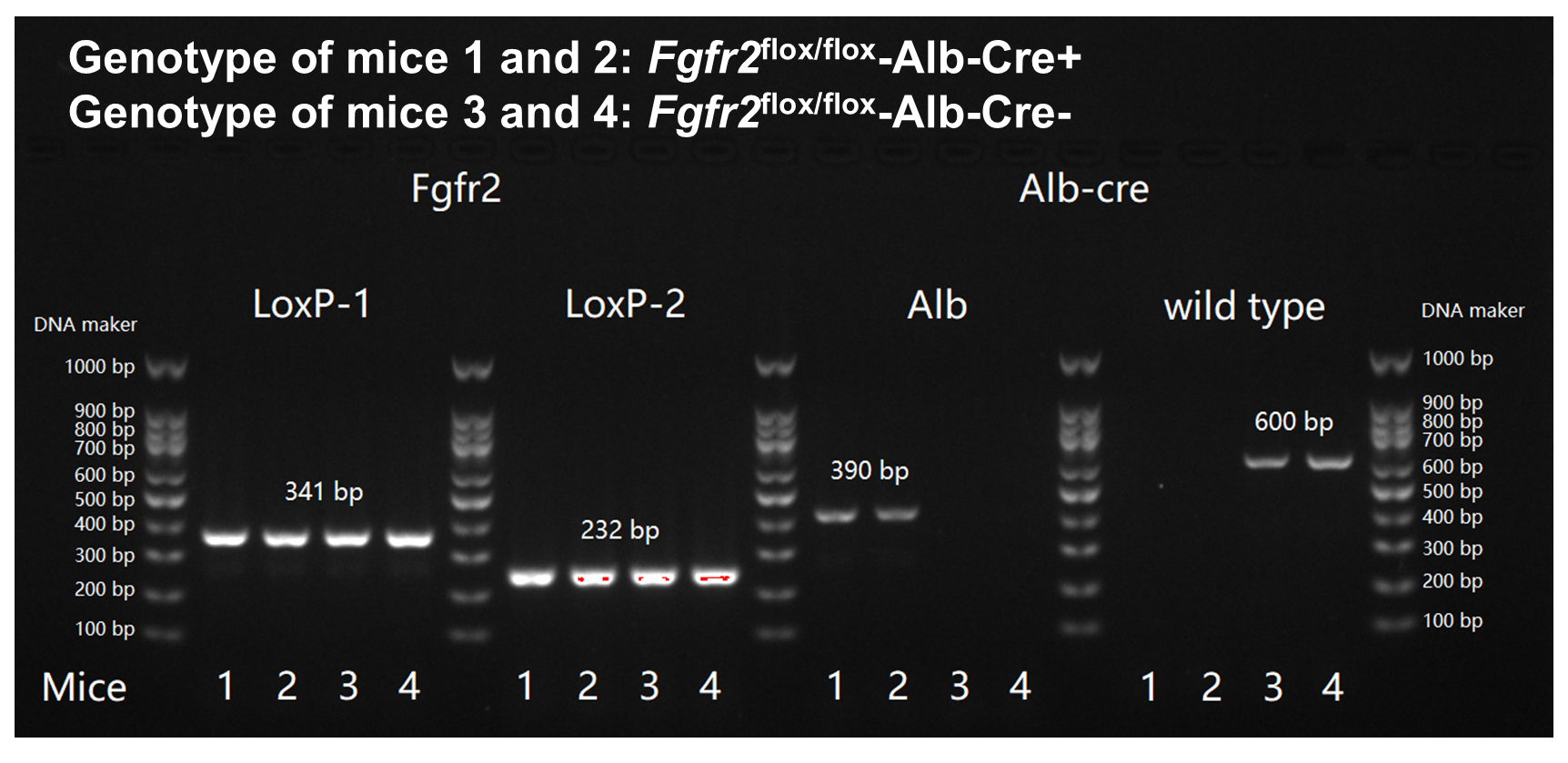
**

**Figure S13. Development of a hepatocyte-specific Fgfr2 knockout model.** PCR genotyping validates the distinction between *Fgfr2^LKO^* and *Fgfr2^FF^* genotypes.

**Figure S14**

**
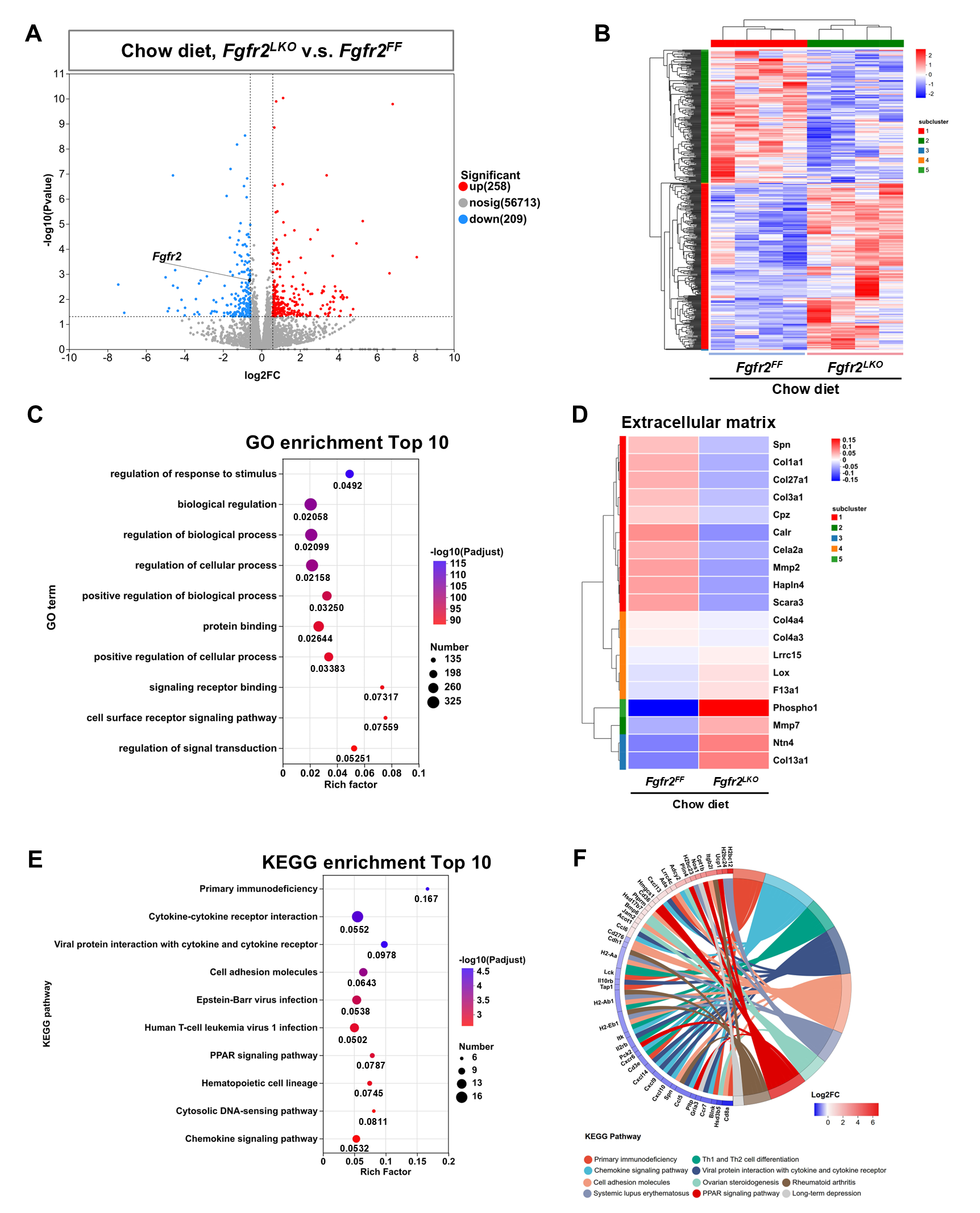
**

**Figure S14. Baseline transcriptomic analysis was conducted to profile hepatocyte-specific deletion of Fgfr2 under chow diet conditions.** (A) A volcano plot illustrates the differentially expressed genes (DEGs) between *Fgfr2^LKO^* and *Fgfr2^FF^* mouse livers under basal conditions. Genes that are significantly upregulated are represented by red dots, significantly downregulated genes by blue dots, and non-significant genes by gray dots (DESeq2 with Benjamini-Hochberg correction; false discovery rate < 0.05; |log2 fold change| ≥ 0.58). (B) An unsupervised hierarchical clustering heatmap displays global gene expression patterns in *Fgfr2^LKO^* and *Fgfr2^FF^* livers, indicating a general similarity between genotypes at baseline. (C) Gene Ontology (GO) enrichment analysis of DEGs identifies the top 10 enriched GO terms, ranked by adjusted P-value. (D) A heatmap of DEGs associated with the GO term "extracellular matrix" reveals modest expression differences in a limited subset of ECM-related genes, without evidence of coordinated profibrotic activation. (E) Kyoto Encyclopedia of Genes and Genomes (KEGG) pathway enrichment analysis of DEGs highlights the top 10 enriched pathways. (F) The chord diagram illustrates the associations between selected DEGs and enriched KEGG pathways, highlighting the distribution of moderate transcriptional changes across various biological pathways rather than their concentration within fibrogenic or inflammatory signaling programs.

**Figure S15**

**
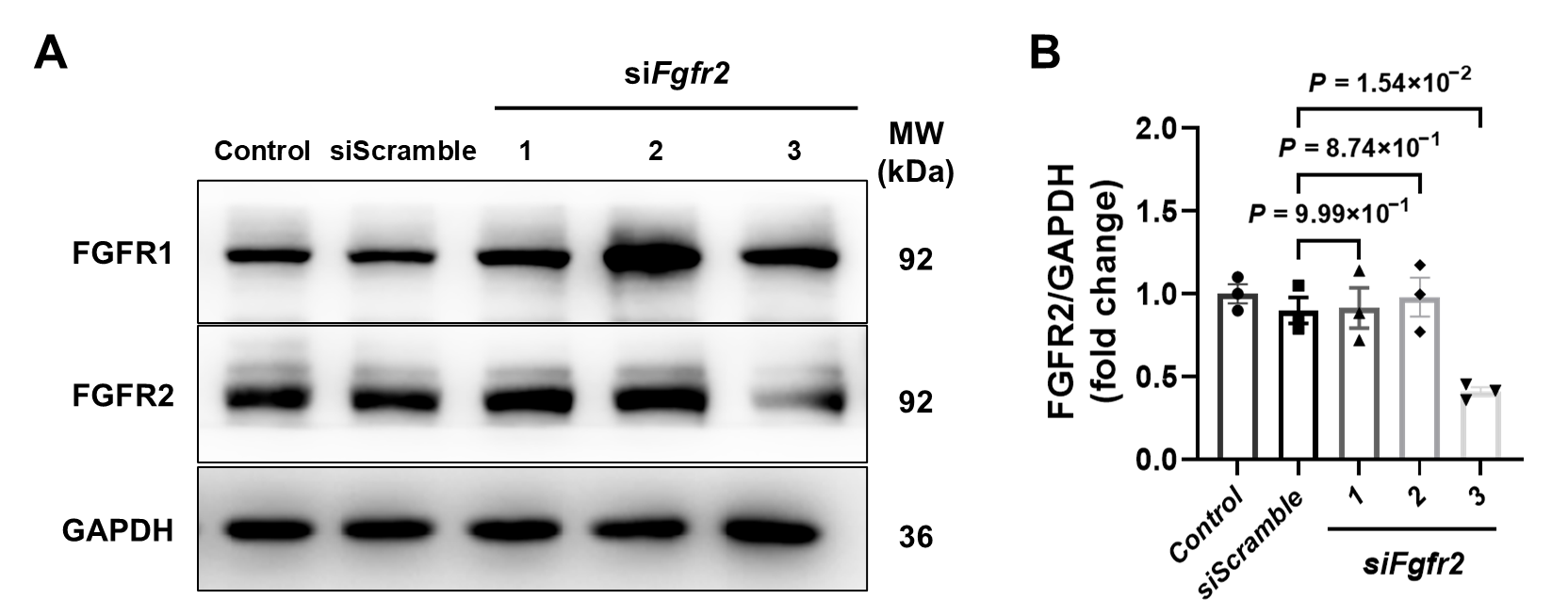
**

**Figure S15. Validation of FGFR2 knockdown in primary mouse hepatocytes.** (A) Primary mouse hepatocytes (PMHs) were transfected with si*Fgfr2* for a duration of 48 hours, after which FGFR2 protein expression was evaluated via immunoblotting. (B) Among the three independent siRNA sequences tested, sequence 3 demonstrated robust and reproducible suppression of FGFR2, and was consequently selected for subsequent experiments. Representative immunoblots and their corresponding quantifications are presented (n = 3). Data are expressed as mean ± SEM and were analyzed using ordinary one-way ANOVA followed by Tukey’s multiple-comparison test. The siRNA sequences utilized are detailed in Supplementary Table S4.

**Figure S16**

**
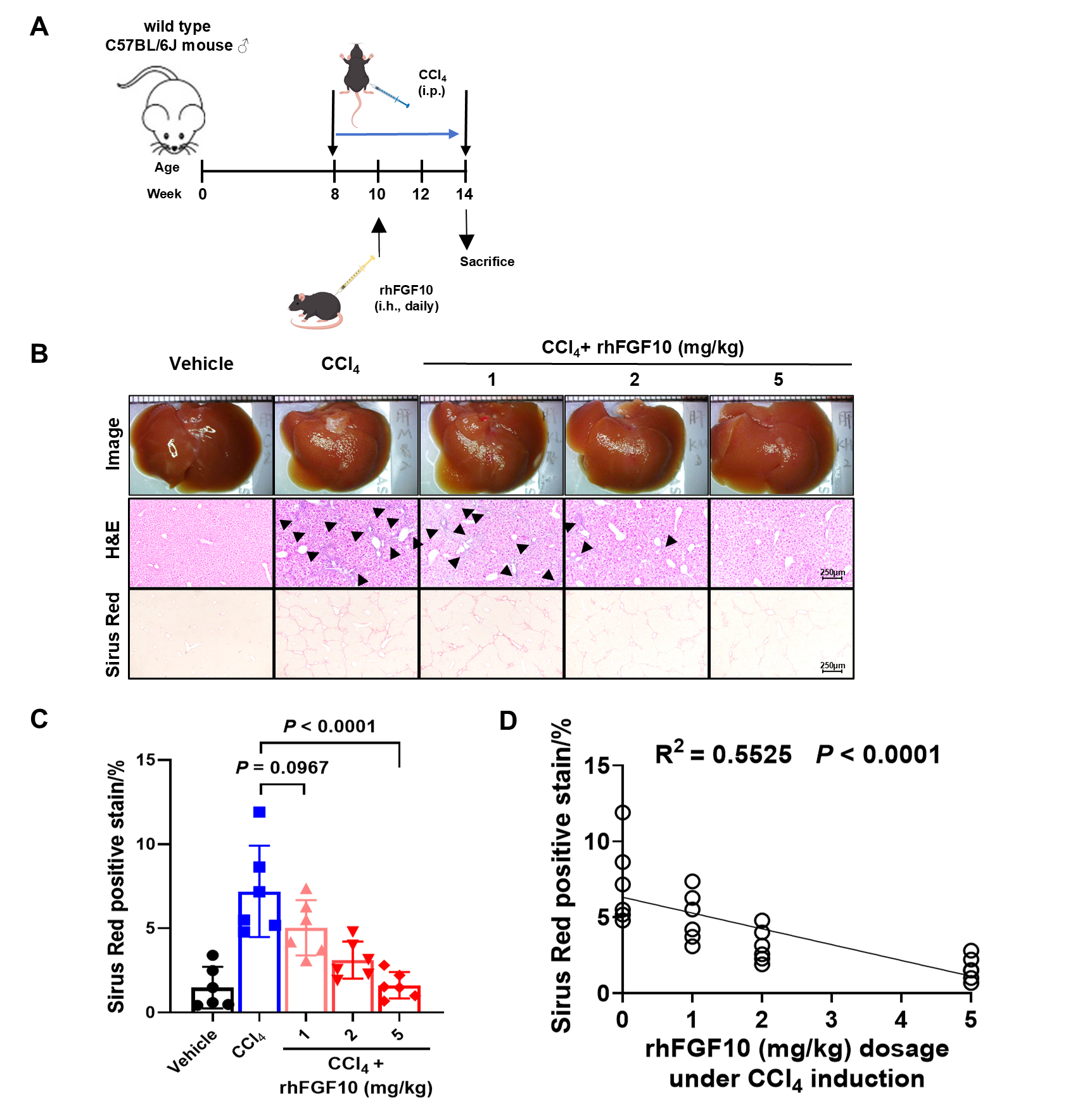
**

**Figure S16. Dose-ranging shows antifibrotic efficacy of rhFGF10 in CCl_4_ mice.** (A) Experimental design: Male C57BL/6J mice, aged 8 weeks, were administered CCl_4_ intraperitoneally, followed by daily subcutaneous injections of rhFGF10 at doses of 1, 2, or 5 mg/kg during the treatment period. (B) Gross liver morphology and histological analysis using H&E and Sirius Red staining were conducted on samples from vehicle-treated, CCl_4_-treated, and CCl_4_ plus rhFGF10-treated groups. Ballooning degeneration is indicated. Scale bar, 250 µm. (C) The percentage area stained by Sirius Red decreased in a dose-dependent manner, with the most significant reduction observed at the 5 mg/kg dose. Data are presented as mean ± SEM, with n = 6 per group. Statistical analysis was performed using one-way ANOVA followed by Dunnett’s test compared to the CCl_4_ group, yielding *P* < 0.0001 for the 5 mg/kg dose and *P* = 0.0967 for the 1 mg/kg dose. (D) Dose-response relationship: Linear regression analysis demonstrated an inverse association between rhFGF10 dose and Sirius Red-stained area.

**Figure S17**

**
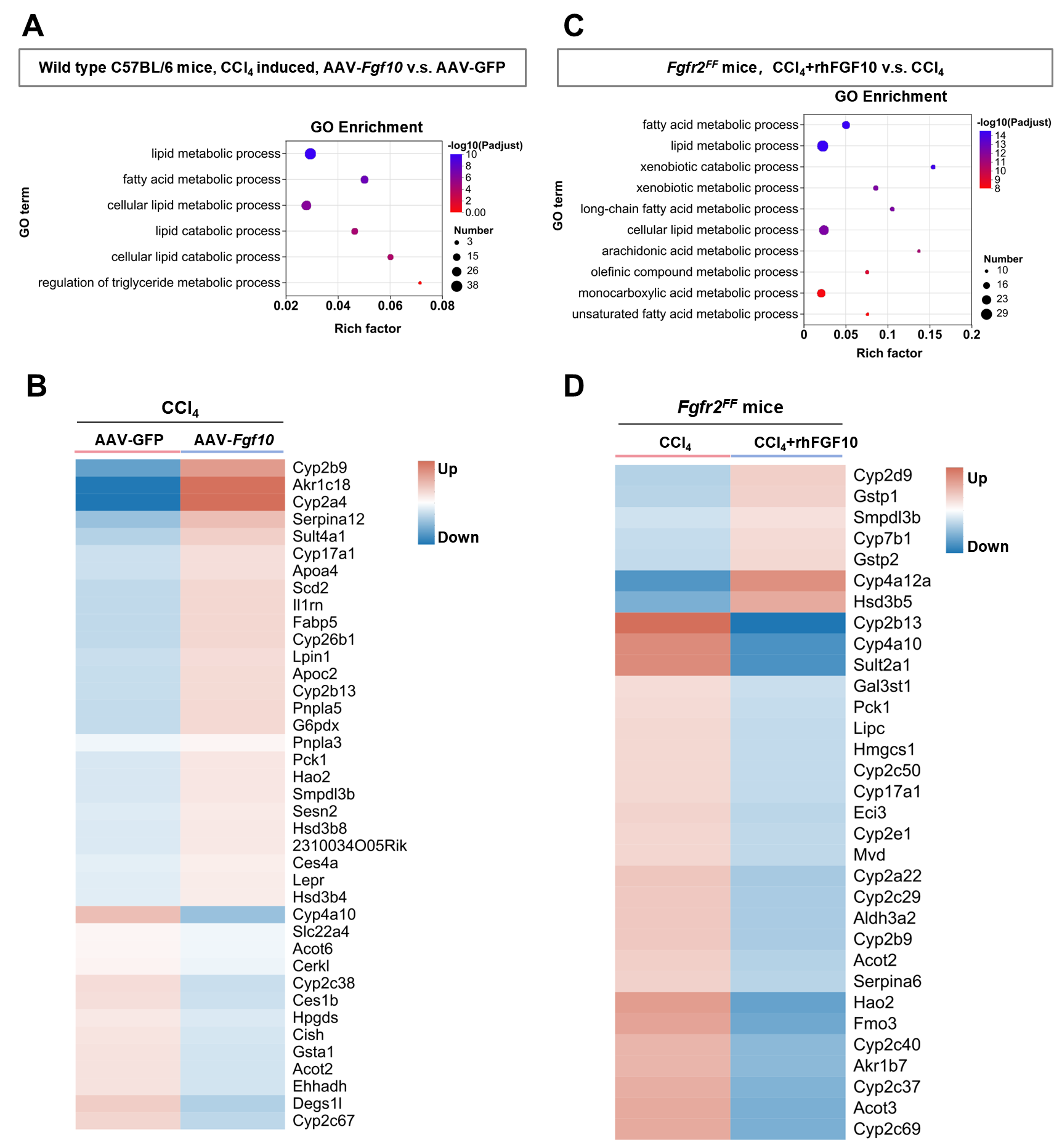
**

**Figure S17. FGF10 influences lipid and fatty acid metabolic pathways in the presence of CCl_4_.** (A-D) Illustrate Gene Ontology (GO) analyses and heatmaps that demonstrate the enrichment of lipid and fatty acid pathways following treatment with AAV-*Fgf10* and rhFGF10, even within a context of non-metabolic injury.

**Supplementary Tables**

**Table S1. Clinical and histological characteristics of MASLD patients included for human liver analysis.**

| Pathology ID | Patient  ID | Steatosis | Lobular inflammation | Ballooning | MASLD activity score | Fibrosis stage | Gender | Age | Serum ALT (U/L) | Serum AST (U/L) | Serum TG (mmol/L) |
| --- | --- | --- | --- | --- | --- | --- | --- | --- | --- | --- | --- |
| D22-44310 | 1607 | 1 | 0 | 0 | 1 | 0 | Male | 39 | 47 | 20 | 3.75 |
| D22-46196 | 1609 | 2 | 2 | 1 | 5 | 0 | Male | 27 | 107 | 65 | 6.17 |
| D22-46318 | 1610 | 0 | 0 | 1 | 1 | 0 | Female | 35 | 30 | 30 | 1 |
| D22-48764 | 1628 | 2 | 1 | 1 | 4 | 0 | Female | 47 | 22 | 21 | 1.54 |
| D22-48790 | 1629 | 3 | 1 | 1 | 5 | 0 | Female | 35 | 34 | 27 | 2.06 |
| D22-51217 | 1662 | 2 | 1 | 0 | 3 | 0 | Female | 35 | 45 | 30 | 3.86 |
| D22-53709 | 1685 | 1 | 0 | 0 | 1 | 0 | Male | 44 | 20 | 12 | 5.5 |
| D18-33379-4 | 784 | 2 | 2 | 0 | 4 | 0 | Female | 32 | 54 | 31 | 7.04 |
| 22-91164 | 1687 | 1 | 0 | 0 | 1 | 0 | Male | 45 | 27 | 16 | 3.01 |
| **Continuation of Table S1** | | | | | | | | | | | |
| D22-56069 | 1688 | 1 | 0 | 0 | 1 | 0 | Female | 32 | 41 | 28 | 2.96 |
| D22-18299 | 1491 | 2 | 2 | 1 | 5 | 1 | Female | 32 | 52 | 34 | 4.17 |
| D22-18440 | 1490 | 1 | 1 | 1 | 3 | 1 | Male | 50 | 33 | 22 | 4.47 |
| D22-37197 | 1574 | 2 | 1 | 1 | 4 | 1 | Female | 38 | 48 | 37 | 1.89 |
| D22-38320 | 1581 | 3 | 2 | 1 | 6 | 1 | Male | 20 | 81 | 62 | 1.89 |
| D22-47557 | 1611 | 2 | 1 | 1 | 4 | 1 | Male | 40 | 114 | 46 | 3.95 |
| D22-30572 | 1534 | 2 | 1 | 1 | 4 | 1 | Male | 18 | 203 | 63 | 1.08 |
| D22-19547 | 1510 | 3 | 2 | 1 | 6 | 1 | Male | 15 | 113 | 44 | 1.95 |
| D22-24440 | 1507 | 2 | 0 | 1 | 3 | 1 | Male | 31 | 27 | 21 | 4.73 |
| D22-43113 | 1606 | 2 | 1 | 1 | 4 | 1 | Female | 28 | 101 | 46 | 2.02 |
| D22-20778 | 1506 | 3 | 2 | 1 | 6 | 2 | Male | 26 | 70 | 41 | 1.29 |
| D22-30781 | 1535 | 2 | 2 | 1 | 5 | 2 | Male | 29 | 248 | 109 | 3.94 |
| D22-39728 | 1591 | 2 | 2 | 1 | 5 | 2 | Male | 53 | 86 | 35 | 3.91 |
| D22-53649 | 1674 | 2 | 1 | 2 | 5 | 2 | Female | 29 | 24 | 34 | 18.79 |
| **Continuation of Table S1** | | | | | | | | | | | |
| D21-09825 | 1271 | 1 | 1 | 1 | 3 | 2 | Female | 28 | 23 | 18 | 1.99 |
| D21-25471-5 | 1333 | 3 | 2 | 2 | 7 | 2 | Male | 18 | 66 | 32 | 1.88 |
| D22-02356 | 1425 | 3 | 2 | 2 | 7 | 2 | Female | 26 | 173 | 121 | 0.92 |
| D20-22646 | 1176 | 3 | 1 | 1 | 5 | 2 | Female | 39 | 84 | 117 | 0.98 |
| A18-17356 | 716 | 2 | 2 | 2 | 6 | 3 | Female | 25 | 660 | 233 | 1.61 |
| 21-71094 | 1373 | 2 | 2 | 2 | 6 | 3 | Male | 66 | 57 | 91 | 2.36 |
| 21-88009 | 1407 | 1 | 1 | 2 | 4 | 3 | Male | 50 | 55 | 26 | 2.34 |
| 23-37271 | 1766 | 2 | 1 | 2 | 5 | 3 | Female | 22 | 41 | 53 | 12.85 |

Formalin-fixed, paraffin-embedded liver tissue samples were procured from patients with Metabolic Associated Steatotic Liver Disease (MASLD) undergoing clinical evaluation at the First Affiliated Hospital of Wenzhou Medical University. The patients were stratified into fibrosis stages 0 (FS0, n = 10), 1 (FS1, n = 9), 2 (FS2, n = 8), and 3 (FS3, n = 4) according to established histological scoring criteria. Comprehensive clinical characteristics, including age, sex, serum alanine aminotransferase (ALT), serum aspartate aminotransferase (AST), metabolic comorbidities, and other clinical variables, are detailed in the accompanying table. The MASLD activity score was assessed semi-quantitatively by summing the scores for steatosis (0-3), lobular inflammation (0-2), and hepatocellular ballooning (0-2), based on histopathological examination of liver sections. All procedures received approval from the Ethics Committee on Clinical Research at the First Affiliated Hospital of Wenzhou Medical University (approval no. 2016/246), and written informed consent was obtained from all participants.

**Table S2. Primers for Genetic Identification of Fgfr2 Liver-Conditional Knockout (*Fgfr2^LKO^*) and Fgfr2 Floxed (*Fgfr2^FF^*) Mice.**

| Item | No. | Forward Sequence | Reverse Sequence |
| --- | --- | --- | --- |
| *Fgfr2-flox/flox* | LoxP-1 | ACGTAAACGGCCACAAGTTC | CTGCTTATTGGGTTCTGCCT |
|  | LoxP-2 | GACATCCTGGGATGCTAAGAATGG | ATCTAGTCCTTAGCTCTCTCCGT |
| *Alb-Cre* | Alb | GAAGCAGAAGCTTAGGAAGATGG | TTGGCCCCTTACCATAACTG |
|  | wild type | GGACAACTTATCCTTATCACAAGGG | TTGGCCCCTTACCATAACTG |

**Note**: The amplicon size produced by the LoxP-1 primer set is 341 base pairs (bp), while the LoxP-2 primer set generates an amplicon of 232 bp. The Alb-Cre primers yield an amplicon size of 390 bp, whereas the amplicon size corresponding to the Alb-Cre wild-type allele is 600 bp.

**Table S3. Antibody information.**

| Antibody | Manufacture | Catalog Number | Dilution | | Application | |
| --- | --- | --- | --- | --- | --- | --- |
| rabbit anti-FGF10 | Bioss | bs-1326R | 1:1000 | | WB | |
| mouse anti-GAPDH | Proteintech | 60004-1-Ig | 1:10000 | | WB | |
| rabbit anti-TGF-β1 | Affinity | AF1027 | 1:1000 | | WB | |
|  |  |  | 1:200 | | IHC | |
| rabbit anti-α-SMA | Bioss | bs-10196R | 1:5000 | | WB | |
|  |  |  | 1:150 | | IHC | |
| rabbit anti-F4/80 | Proteintech | 28463-1-AP | 1:5000 | | IHC | |
| mouse anti-TNFα | Proteintech | 60291-1-Ig | 1:5000 | | WB | |
| rabbit anti-IL-1β | Bioss | bs-0812R | 1:1000 | | WB | |
| rabbit anti-IL6 | Bioss | bs-6309R | 1:1000 | | WB | |
| rabbit anti-Col1a1 | ABclonal | A1352 | 1:1500 | | WB | |
| rabbit anti-Col3a1 | ABclonal | A3795 | 1:1500 | | WB | |
| rabbit anti-phospho-NF-κB p65 (Ser468) | Proteintech | 82335-1-RR | 1:5000 | | WB | |
| **Continuation of Table S3** | | | | | | |
| mouse anti-NF-κB p65 | Proteintech | 66535-1-Ig | | 1:2000 | | WB |
| mouse anti-IkB Alpha | Proteintech | 66418-1-Ig | | 1:20000 | | WB |
| rabbit anti-FGFR1 | Abclonal | A0082 | | 1:1000 | | WB |
| rabbit anti-FGFR2 | Abclonal | A12436 | | 1:1000 | | WB |
|  |  |  |  | 1:200 | | IF |
| rabbit anti-phospho-FRS2-α (Tyr196) | Cell Signaling Technology | 3864 | | 1:1000 | | WB |
| rabbit anti-FRS2α | Proteintech | 11503-1-AP | | 1:2000 | | WB |
| mouse anti-phospho-GSK3β (Ser9) | Proteintech | 67558-1-Ig | | 1:2000 | | WB |
| rabbit anti-GSK3B | Proteintech | 22104-1-AP | | 1:2000 | | WB |
| mouse anti-Albumin | Proteintech | 66051-1-Ig | | 1:20000 | | WB |
| rabbit anti-FGF10 AF488 conjugated | Signalway Antibody | C32224-AF488 | | 1:200 | | IF |
| rabbit anti-Desmin AF647 conjugated | Signalway Antibody | C33355-AF647 | | 1:200 | | IF |
| rabbit anti-α-SMA AF555 conjugated | Signalway Antibody | C48499-AF555 | | 1:200 | | IF |
| rabbit anti-FGF10 AF488 conjugated | Signalway Antibody | C32224-AF488 | | 1:200 | | IF |
| rabbit anti-Desmin AF647 conjugated | Signalway Antibody | C33355-AF647 | | 1:200 | | IF |
| **Continuation of Table S3** |  |  |  | |  | |
| CoraLite647-conjugated AffiniPure F(ab')2 Fragment Goat Anti-Mouse IgG (H+L) | Proteintech | SA00014-10 | 1:100 | | IF | |
| Phospho-FGFR2 (Ser782) Antibody | Affinity | AF8437 | 1:1000 | | WB | |
| HRP-conjugated Affinipure Goat Anti-Mouse IgG | Proteintech | SA00001-1 | 1:10000 | | WB | |
| HRP-conjugated Affinipure Goat Anti-Rabbit IgG | Proteintech | SA00001-2 | 1:10000 | | WB | |

**Note:** IHC, immunohistochemistry; WB, Western Blot; IF, immunofluorescence; FGF10, fibroblast growth factor 10; GAPDH, glyceraldehyde-3-phosphate dehydrogenase; TGF-β1, transforming growth factor-β1; α-SMA, alpha-smooth muscle actin; F4/80, adhesion G protein-coupled receptor E1 (ADGRE1); TNFα, tumor necrosis factor alpha; IL-1β, interleukin-1 beta; IL-6, interleukin-6; Col1a1, type I collagen alpha-1 chain; Col3a1, type III collagen alpha-1 chain; NF-κB, nuclear factor kappa B; IκBα, inhibitor of NF-κB alpha; FGFR1, fibroblast growth factor receptor 1; FGFR2, fibroblast growth factor receptor 2; FRS2α, fibroblast growth factor receptor substrate 2 alpha; GSK3β, glycogen synthase kinase 3β; HRP, horseradish peroxidase.

**Table S4. Sequences of siRNAs targeting mouse *Fgfr2*.**

| Name | No. | Forward Sequence | Reverse Sequence |
| --- | --- | --- | --- |
| Mouse si*Ffgfr2* | 1 | GGUCCAUCAACCACACCUATT | UAGGUGUGGUUGAUGGACCTT |
|  | 2 | CAGUGGGAAUCGAUAAAGATT | UCUUUAUCGAUUCCCACUGTT |
|  | 3 | CUCUACGUCAUAGUUGAAUTT | AUUCAACUAUGACGUAGAGTT |

**Note:** This table presents the nucleotide sequences of small interfering RNAs (siRNAs) employed for the specific silencing of fibroblast growth factor receptor 2 (*Fgfr2*; Gene ID: 14183) in primary mouse hepatocytes. The *siFgfr2* sequences are designed to target the mRNA of the mouse *Fgfr2* gene. These sequences were synthesized and subsequently validated for their knockdown efficiency in primary mouse hepatocytes, with the aim of evaluating the functional role of FGFR2 signaling in downstream cellular responses.

**Table S5. Primer sequences used for quantitative real-time PCR (qRT-PCR) analysis of target genes.**

| Gene | ID | Forward Sequence | Reverse Sequence |
| --- | --- | --- | --- |
| Mouse *Gapdh* | 14433 | AGGTCGGTGTGAACGGATTTG | TGTAGACCATGTAGTTGAGGTCA |
| Mouse Fgf10 | 14165 | TCAGCGGGACCAAGAATGAAG | CGGCAACAACTCCGATTTCC |
| Mouse Fgfr2 | 14183 | AATCTCCCAACCAGAAGCGTA | CTCCCCAATAAGCACTGTCCT |
| Mouse *Fasn* | 14104 | CTGTGCCCGTCGTCTATACC | AACCTGAGTGGATGAGCACG |
| Mouse *Srebf1* | 20787 | TGACCCGGCTATTCCGTGA | CTGGGCTGAGCA ATACAGTTC |
| Mouse *Mvk* | 17855 | GGTGTGGTCGGAACTTCCC | CCTTGAGCGGGTTGGAGAC |
| Human *GAPDH* | 2597 | ACAACTTTGGTATCGTGGAAGG | GCCATCACGCCACAGTTTC |
| Human *FGF1* | 2246 | GCCCTGACCGAGAAGTTTAATC | CCCCGTTGCTACAGTAGAGG |
| Human *FGF2* | 2247 | AGAAGAGCGACCCTCACATCA | CGGTTAGCACACACTCCTTTG |
| Human *FGF4* | 2249 | CTCGCCCTTCTTCACCGATG | GTAGGACTCGTAGGCGTTGTA |
| Human *FGF5* | 2250 | AGAGTGGGCATCGGTTTCCATC | CCTACAATCCCCTGAGACACAG |
| Human *FGF6* | 2251 | AACAACACGCTGCTGGACT | GATGCCCACGTTGCAGTAGA |
| Human *FGF7* | 2252 | CTGTCGAACACAGTGGTACCTG | CCAACTGCCACTGTCCTGATTTC |
| Human *FGF8* | 2253 | AGGGGAAGCTAATTGCCAAGA | CCTTGCGGGTAAAGGCCAT |
| Human *FGF9* | 2254 | ATGGCTCCCTTAGGTGAAGTT | CCCAGGTGGTCACTTAACAAAAC |
| Human FGF10 | 2255 | CAGTAGAAATCGGAGTTGTTGCC | TGAGCCATAGAGTTTCCCCTTC |
| **Continuation of Table S5** | | | |
| Human *FGF11* | 2256 | CTGTACGCCTCTGCTCTCTAC | GCCTTGGTCTTCTTAACTCGGT |
| Human *FGF12* | 2257 | CCAGCAAGAATCAGGCCGAG | TCGGTACAAAATGTGATGAGGG |
| Human *FGF13* | 2258 | GTTACCAAGCTATACAGCCGAC | ACAGGGATGAGGTTAAACAGAGT |
| Human *FGF14* | 2259 | GGAAGGGCAAGCTATGAAAGG | TGGTTCTCGGTACATGGCAAC |
| Human *FGF16* | 8823 | TATGGGTCGAAGAAACTCACACG | TGAGGCATAGGTGTTGTACCAG |
| Human *FGF17* | 8822 | AAGACTGCGTGTTCACGGAG | CAAACTCGAACTGCTTCTGCT |
| Human *FGF18* | 8817 | ACTTGCCTGTGTTTACACTTCC | GACCTGGATGTGTTTCCCACT |
| Human *FGF19* | 9965 | CGGAGGAAGACTGTGCTTTCG | CTCGGATCGGTACACATTGTAG |
| Human *FGF20* | 26281 | ATGGCTCCCTTAGCCGAAGT | AGGAAATGCGAACCCACCTG |
| Human *FGF21* | 26291 | GCCTTGAAGCCGGGAGTTATT | GTGGAGCGATCCATACAGGG |
| Human *FGF22* | 27006 | GGGAGCGCATCGAAGAGAAC | CTGTGAGGCGTAGGTGTTGTG |
| Human *FGF23* | 8074 | CAGAGCCTATCCCAATGCCTC | GGCACTGTAGATGGTCTGATGG |

**Note:** This table presents the sequences of forward and reverse primers employed in the quantitative reverse transcription polymerase chain reaction (qRT-PCR) analysis of gene expression in murine liver tissue and primary murine cells, as well as in human liver samples. The target genes include murine *Fgf10*, *Fgfr2*, *Fasn*, *Srebf1*, *Mvk*, and *Gapdh* (serving as the internal control), in addition to human *GAPDH* and *FGF1* through *FGF23* (noting that *FGF15* is absent in humans, with *FGF19* being the human ortholog). It is noteworthy that human *FGF3* was not detected in our qRT-PCR assays.

**Table S6. List of abbreviations.**

| Abbreviations | Full name |
| --- | --- |
| MASLD | metabolic dysfunction-associated steatotic liver disease |
| MASH | metabolic dysfunction-associated steatohepatitis |
| FS | fibrosis stage |
| NAS | MASLD activity score |
| IF | immunofluorescence |
| snRNA-seq | Single-nucleus RNA sequencing |
| UMAP | Uniform Manifold Approximation and Projection |
| SPF | specific pathogen-free |
| FGF10 | fibroblast growth factor 10 |
| FGFR | fibroblast growth factor receptor |
| FGFR1 | fibroblast growth factor receptor 1 |
| FGFR2 | fibroblast growth factor receptor 2 |
| HFD | high-fat diet |
| AAV | adeno-associated virus |
| rhFGF10 | recombinant human fibroblast growth factor 10 |
| FBS | fetal bovine serum |
| AST | aspartate aminotransferase |
| ALT | alanine aminotransferase |
| DMEM | Dulbecco's modified eagle medium |
| HSCs | hepatic stellate cells |
| H&E | hematoxylin and eosin |
| SR | Sirius Red |
| CCl_4_ | carbon tetrachloride |
| IHC | immunohistochemistry |
| IF | immunofluorescence |
| TGFβ1 | transforming growth factor-beta 1 |
| IL-1β | interleukin-1 beta |
| IL-6 | interleukin- 6 |
| **Continuation of Table S6** | |
| TNF-α | tumor necrosis factor alpha |
| α-SMA | alpha smooth muscle actin |
| F4/80 | adhesion G protein-coupled receptor E1 |
| RNA-seq | RNA sequencing |
| Col1α1 | type I collagen alpha-1 chain (protein) |
| Col3α1 | type III collagen alpha-1 chain (protein) |
| Col1α1 | collagen type I alpha-1 chain (gene) |
| Col3α1 | collagen type III alpha-1 chain (gene) |
| Col5a2 | collagen type V alpha-2 chain (gene) |
| ECM | extracellular matrix |
| FITC | fluorescein isothiocyanate |
| PI | propidium iodide |
| NF-κB | nuclear factor kappa B |
| IκBα | inhibitor of NF-κB alpha |
| TUNEL | terminal deoxynucleotidyl transferase dUTP nick-end labeling |
| FRS2α | fibroblast growth factor receptor substrate 2 alpha |
| GSK3β | glycogen synthase kinase 3β |
| Erbb3 | receptor tyrosine-protein kinase erbB-3 |
| Epha7 | ephrin type-A receptor 7 |
| Pdgfra | platelet-derived growth factor receptor alpha |
| Epha3 | ephrin type-A receptor 3 |
| Fgfr3 | fibroblast growth factor receptor 3 |
| Ntrk2 | BDNF/NT-3 growth factors receptor |
| Erbb4 | receptor tyrosine-protein kinase erbB-4 |
| PCA | principal component analysis |
| qRT-PCR | quantitative reverse transcription polymerase chain reaction |
| DEGs | differentially expressed genes |
| GO | Gene Ontology |
| KEGG | Kyoto Encyclopedia of Genes and Genomes |
| **Continuation of Table S6** | |
| SDS-PAGE | sodium dodecyl sulfate polyacrylamide gel electrophoresis |
| siRNA | small interfering RNA |
| WB | Western blot |
| PVDF | polyvinylidene difluoride |
| OCT | optimal cutting temperature compound |
| DAB | 3,3′-diaminobenzidine |
| PBS | phosphate-buffered saline |
| BSA | bovine serum albumin |
| DAPI | 4′,6-diamidino-2-phenylindole |
| KRT | Key Resources Table |
