# Supplementary material for: Fibroblast growth factor 10 attenuates advanced liver fibrosis through hepatocyte fibroblast growth factor receptor 2 signalling
Source: Clin Transl Med. 2026 May 12;16(5):e70675. doi: 10.1002/ctm2.70675 (PMC13162133; doi:10.1002/ctm2.70675)
Supplement: Supplementary file 2 — Supporting Information [file CTM2-16-e70675-s002.docx]

**KEY RESOURCES TABLE**

| REAGENT or RESOURCE | SOURCE | IDENTIFIER (Cat#, clone) |
| --- | --- | --- |
| Antibodies |  |  |
| Rabbit anti-FGF10 | Bioss | bs-1326R; RRID: N/A |
| Rabbit anti-FGF7 | Proteintech | 10939-1-AP; RRID: AB_2102816 |
| Mouse anti-GAPDH | Proteintech | 60004-1-Ig; RRID: AB_2107436 |
| Rabbit anti-TGF-β1 | Affinity | AF1027; RRID: AB_2835389 |
| Rabbit anti-α-SMA | Bioss | bs-10196R; RRID: N/A |
| Rabbit anti-F4/80 | Proteintech | 28463-1-AP; RRID: AB_2881149 |
| Mouse anti-TNFα | Proteintech | 60291-1-Ig; RRID: AB_2833255 |
| Rabbit anti-IL-1β | Proteintech | 26048-1-AP; RRID: AB_2880351 |
| Rabbit anti-IL-6 | Proteintech | 21865-1-AP; RRID: AB_11142677 |
| Rabbit anti-Col1a1 | ABclonal | A1352; RRID: AB_2760381 |
| Rabbit anti-Col3a1 | ABclonal | A3795; RRID: AB_2765308 |
| Rabbit anti-phospho-NF-κB p65 (Ser468) | Proteintech | 82335-1-RR; RRID: AB_3083091 |
| Mouse anti-NF-κB p65 | Proteintech | 66535-1-Ig; RRID: AB_2881898 |
| Mouse anti-IκBα | Proteintech | 66418-1-Ig; RRID: AB_2881790 |
| Rabbit anti-FGFR1 | ABclonal | A0082; RRID: N/A |
| Rabbit anti-FGFR2 | ABclonal | A12436; RRID: N/A |
| Rabbit anti-phospho-FRS2α (Tyr196) | Cell Signaling Technology | 3864; RRID: N/A |
| Rabbit anti-FRS2α | Proteintech | 11503-1-AP; RRID: AB_2262942 |
| Mouse anti-phospho-GSK3β (Ser9) | Proteintech | 67558-1-Ig; RRID: AB_2882772 |
| Rabbit anti-GSK3β | Proteintech | 22104-1-AP; RRID: AB_2878997 |
| Mouse anti-Albumin | Proteintech | 66051-1-Ig; RRID: AB_11042320 |
| Rabbit anti-FGF10-AF488 | Signalway | C32224-AF488; RRID: N/A |
| Rabbit anti-Desmin-AF647 | Signalway | C33355-AF647; RRID: N/A |
| Rabbit anti-α-SMA-AF555 | Signalway | C48499-AF555; RRID: N/A |
| Goat anti-Mouse IgG (H+L), F(ab’)2, CoraLite647 | Proteintech | SA00014-10; RRID: N/A |
| Phospho-FGFR2 (Ser782) | Affinity | AF8437; RRID: AB_2840494 |
| HRP-Goat anti-Mouse IgG | Proteintech | SA00001-1; RRID: AB_2722565 |
| HRP-Goat anti-Rabbit IgG | Proteintech | SA00001-2; RRID: AB_2722564 |
| Chemicals and reagent |  |  |
| Recombinant human FGF10 (rhFGF10) | National Engineering Research Center for Cell Growth Factor Drugs and Protein Preparations, Wenzhou Medical University, Wenzhou, Zhejiang, CHINA | bioactivity 2.8×10^5 AU/mg; <5 EU/mg endotoxin |
| Rodent Diet With 60 kcal% Fat | Research Diets | D12492 |
| Carbon tetrachloride, AR | Sinopharm Chemcial Reagent | 10006464 |
| Olive oil | Aladdin | O108686 |
| Collagenase, Type IV | Thermo Fisher Scientific | 17104019 |
| Fetal Bovine Serum, qualified, Brazil | Thermo Fisher Scientific | 10270106 |
| Lipofectamine™ 3000 Transfection Reagent | Thermo Fisher Scientific | L3000015 |
| Axis-Shield Density Gradient Media (OptiPrep™ 250 mL) | Serumwerk Bernburg AG | AXS-1114542 |
| DAPI Fluoromount-GTM | YEASEN | 36308ES20 |
| Triton X-100 | Aladdin | T109026 |
| Collagen I，rat Tail(liquid) | Solarbio | C8065 |
| Paraformaldehyde (4%) | Solarbio | P1110 |
| Triquick Reagent  (Trizol Substitute) | Solarbio | R1100 |
| Normal Goat Serum | Solarbio | SL038 |
| Critical commercial assays |  |  |
| Glutamic-oxalacetic Transaminase (GOT/AST) Assay Kit | Solarbio | BC1565 |
| Glutamic-pyruvic Transaminase (GPT) Activity Assay stain Kit | Solarbio | BC1555 |
| Hematoxylin-Eosin (HE) Stain Kit | Solarbio | G1120 |
| Modified Oil Red O Stain Kit | Solarbio | G1263 |
| Sirius Red stain kits | Huayueyang | GH6044plus |
| One-Step TUNEL Apoptosis Kit (Red) | Beyotime | C1089 |
| Annexin V-FITC/PI Kit | MeilunBio | MA0220 |
| Super ECL Detection Reagent ECL | YEASEN | 36208ES60 |
| BCA Protein Quantification Kit | Vazyme | E112 |
| cDNA Synthesis Kit | Vazyme | R212-01 |
| SYBR qPCR Master Mix | Vazyme | Q711 |
| SABC-POD (rabbit IgG) DAB kit (for IHC) | Wuhan Boster | SA1022 |
| Biological samples-human |  |  |
| Human liver biopsies (MASLD, stratified FS0-FS3) | First Affiliated Hospital of Wenzhou Medical University | Ethics 2016/246 |
| Experimental models: Organisms/strains |  |  |
| C57BL/6J male mice  (5-8 weeks) | gempharmatech | Strain NO. N000013 |
| *Fgfr2*^flox/flox^ mice | Cyagen | S-CKO-02415 |
| Alb-Cre mice | Cyagen | C00106 |
| *Fgfr2^LKO^* (Alb-Cre; *Fgfr2*^flox/flox^ ) | This study | PCR genotyping (Table S2) |
| Experimental models: Cell lines / primary cells |  |  |
| Primary mouse hepatocytes | This study | N/A |
| Primary mouse hepatic stellate cells (HSCs) | This study | N/A |
| Viral vectors |  |  |
| AAV2/8-CAG-Fgf10 | OBiO Technology (Shanghai) | HYKY-220419048-DAAV |
| AAV2/8-CAG-GFP (control) | OBiO Technology (Shanghai) | HYKY-220419048-DAAV |
| Oligonucleotides |  |  |
| DNA primers or oligos, see Table S2 and S5 | Sangon Biotech | N/A |
| siRNA, see Table S4 | OBiO Technology | N/A |
| Software and algorithms |  |  |
| GraphPad Prism 9 | GraphPad Software, LLC (Dotmatics) | https://www.graphpad.com/features; RRID: SCR_002798 |
| Image-Pro Plus 7.0 | Media Cybernetics | https://mediacy.com/image-pro/; RRID:SCR_007369 |
| Zeiss ZEN 3.6 | Carl Zeiss Microscopy GmbH | https://www.zeiss.com/microscopy/us/products/software/zeiss-zen.html; RRID:SCR_013672 |
| Nikon NIS-Elements v5.30 | Nikon Instruments Inc. | https://www.microscope.healthcare.nikon.com/products/software/nis-elements; RRID:SCR_014329 |
| Living Image (IVIS) 4.4 | Revvity（PerkinElmer） | https://www.revvity.com/category/in-vivo-imaging-software; RRID:SCR_014247 |
| Fastp v0.24.0 | OpenGene / Shifu Chen | https://github.com/OpenGene/fastp; RRID:SCR_016962 |
| HiSat2 v2.2.1 | Daehwan Kim Lab, Johns Hopkins | http://ccb.jhu.edu/software/hisat2/index.shtml; RRID:SCR_015530 |
| StringTie v2.2.0 | Center for Computational Biology, Johns Hopkins University (Pertea/Salzberg labs) | https://ccb.jhu.edu/software/stringtie/; RRID:SCR_016323 |
| RSEM v1.3.3 | Dewey Lab (UW-Madison) | http://deweylab.github.io/RSEM/; RRID:SCR_013027 |
| DESeq2 v1.42.0 | Bioconductor | http://bioconductor.org/packages/stats/bioc/DESeq2/; RRID:SCR_015687 |
| GOATOOLS v1.4.4 | GOATOOLS developers / PyPI | https://github.com/tanghaibao/GOatools; RRID:SCR_025305 |
| Python scipy | SciPy community | https://scipy.org/install/; RRID:SCR_008058 |
